# The diversity and biogeography of the Coleoptera of Churchill: insights from DNA barcoding

**DOI:** 10.1186/1472-6785-13-40

**Published:** 2013-10-29

**Authors:** Thomas S Woodcock, Elizabeth E Boyle, Robert E Roughley, Peter G Kevan, Renee N Labbee, Andrew B T Smith, Henri Goulet, Dirk Steinke, Sarah J Adamowicz

**Affiliations:** 1 School of Environmental Science, University of Guelph, 50 Stone Rd. E., Guelph, ON, Canada; 2 Biodiversity Institute of Ontario & Department of Integrative Biology, University of Guelph, 50 Stone Rd. E., Guelph, ON, Canada; 3 Department of Entomology, University of Manitoba, Winnipeg, MB, Canada; 4 Canadian Museum of Nature, P.O. Box 3443, Station D, Ottawa, ON, Canada; 5 Canadian National Collection, 960 Carling Ave., Ottawa, ON, Canada

**Keywords:** Barcode library, Barcoding biotas, Boreal-arctic transition, COI, Cytochrome *c* oxidase subunit I, DNA barcoding, Freshwater, Terrestrial, Subarctic forest

## Abstract

**Background:**

Coleoptera is the most diverse order of insects (>300,000 described species), but its richness diminishes at increasing latitudes (e.g., ca. 7400 species recorded in Canada), particularly of phytophagous and detritivorous species. However, incomplete sampling of northern habitats and a lack of taxonomic study of some families limits our understanding of biodiversity patterns in the Coleoptera. We conducted an intensive biodiversity survey from 2006–2010 at Churchill, Manitoba, Canada in order to quantify beetle species diversity in this model region, and to prepare a barcode library of beetles for sub-arctic biodiversity and ecological research. We employed DNA barcoding to provide estimates of provisional species diversity, including for families currently lacking taxonomic expertise, and to examine the guild structure, habitat distribution, and biogeography of beetles in the Churchill region.

**Results:**

We obtained DNA barcodes from 3203 specimens representing 302 species or provisional species (the latter quantitatively defined on the basis of Molecular Operational Taxonomic Units, MOTUs) in 31 families of Coleoptera. Of the 184 taxa identified to the level of a Linnaean species name, 170 (92.4%) corresponded to a single MOTU, four (2.2%) represented closely related sibling species pairs within a single MOTU, and ten (5.4%) were divided into two or more MOTUs suggestive of cryptic species. The most diverse families were the Dytiscidae (63 spp.), Staphylinidae (54 spp.), and Carabidae (52 spp.), although the accumulation curve for Staphylinidae suggests that considerable additional diversity remains to be sampled in this family. Most of the species present are predatory, with phytophagous, mycophagous, and saprophagous guilds being represented by fewer species. Most named species of Carabidae and Dytiscidae showed a significant bias toward open habitats (wet or dry). Forest habitats, particularly dry boreal forest, although limited in extent in the region, were undersampled.

**Conclusions:**

We present an updated species list for this region as well as a species-level DNA barcode reference library. This resource will facilitate future work, such as biomonitoring and the study of the ecology and distribution of larvae.

## Background

Coleoptera is the most diverse order of insects in the world [1] and dominates many ecosystems in terms of individual abundance and niches occupied. In Canada, there are >7400 described species of beetles in approximately 112 families [2-5]. Beetle richness diminishes at increasing latitudes, and only 14% of Canadian beetle families occur north of the tree line [6]. Many northern areas, however, remain chronically undercollected due to logistical limitations. Danks [7] reported 167 named species in 18 families from the arctic north of the tree line. Anderson [4] reported 913 species from the Yukon, with an additional 822 found in adjacent regions of Alaska and the Northwest Territories. Canadian insect richness tends to be greater in the west, although in the Churchill region of northeastern Manitoba, Canada, the transition between boreal forest and open-ground habitats results in increased habitat heterogeneity, and this region is of great importance for understanding postglacial insect distributions [3,8-10]. Furthermore, recolonization of the landscape following the Last Glacial Maximum (LGM) potentially included arctic species from the north and west (Beringia) as well as from the south as the ice receded.

Danks [3] suggests that the importance of competition for food resources in structuring communities decreases with increasing latitude, and abiotic (climatic) factors are the major drivers of northern biodiversity. Insect species show a stronger response to latitude than longitude, such that boreal and tundra species tend to occur across the continent according to their habitat [3,6]. It is generally expected that boreal forest species are likely to be transcontinental in distribution, while tundra and high arctic species colonized from refugia in the west and north following deglaciation. Following the LGM approximately 8000 years ago in the Churchill region [11], recolonization of flora and fauna proceeded from southern and western species pools, and species from refugia in the south, north, and in Beringia could potentially be present [3,6,10,12]. Furthermore, eastern tundra species would not be expected to disperse to the area due to the obstacle of Hudson Bay, although eastern boreal forest species may be present [3]. Schwert & Ashworth [12] suggest that Beringia was the dominant refugium for northern Coleoptera species, which spread eastward following the glacial retreat from the Arctic coast. Garry [10], however, maintains that many of these species are well represented in fossil assemblages in the United States Midwest, and subsequently expanded northward. More recently, climate change and the warming of the Churchill River could represent forces accelerating the arrival of southern beetle species [13], particularly aquatic species that are strong dispersers.

The value of a regional faunal approach to biodiversity study is now well established and provides a means of rapid assessment of the diversity of traditionally understudied groups, together with the ready dissemination of taxon-specific data via DNA barcode libraries [14-19]. In previous surveys of the Churchill region, McClure [20] reported 62 total species in 20 families of Coleoptera in his surveys of both terrestrial and aquatic habitats near Churchill. Garry [10] surveyed carabid beetles in the region west and northwest of Churchill and reported 65 species. In the north, the majority of species are predaceous, with smaller numbers of species representing phytophagous and saprophagous guilds [3,7]. An intensive biodiversity survey conducted from 2006–2010 in the Churchill region introduced in [18] revealed that Hymenoptera [21] and Diptera (Wang et al. unpubl.) are more diverse than Coleoptera in the sub-arctic region of Churchill, while Lepidoptera (deWaard et al., unpubl.) and Coleoptera (this study) have similar levels of diversity.

This paper presents a DNA barcode library of the Churchill regional beetle fauna and examines patterns of diversity in terms of taxonomy and ecology. As this is a small-scale regional study, DNA barcoding is expected to have high effectiveness for separating beetle species [22]. This library represents a valuable resource for researchers in the future, both in this region and in other parts of the north, particularly in systematic, biogeographic, and ecological studies; in research requiring larval identifications; and in monitoring potential faunal changes related to climate change.

## Methods

### Field collections, specimen selection, and identification

**Figure 1:**
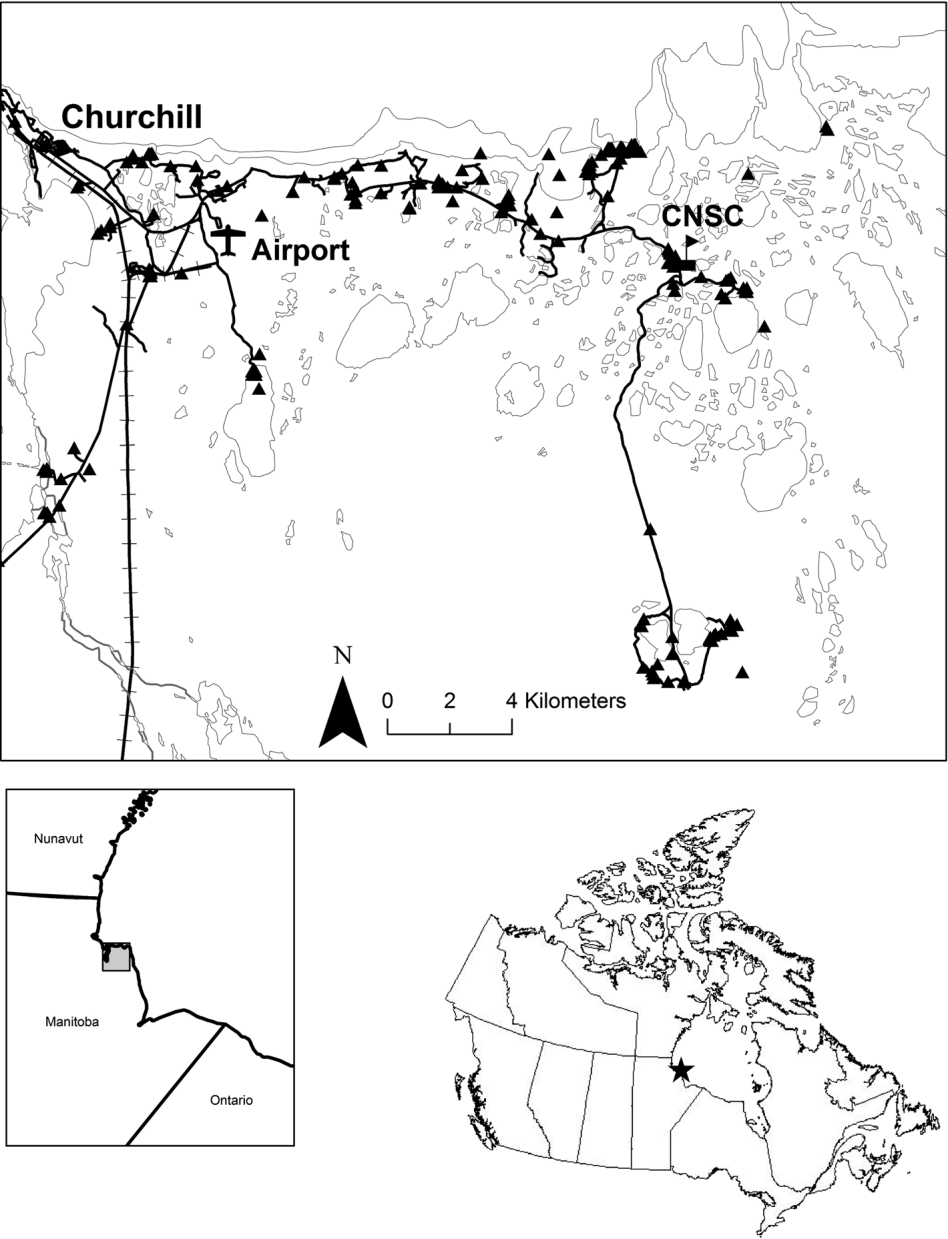
**Map of specimen collection sites of Coleoptera in the Churchill Region, Manitoba.** Several additional sites in northeastern Manitoba beyond the mapped Churchill region are also included in analysis. CNSC = Churchill Northern Studies Center. Insets show the coverage of the map in Manitoba (lower left), and the location within Canada (lower right).

Adult terrestrial beetle specimens were collected between 2006 and 2010 at 223 sites in the Churchill region (Figure 1) primarily using pitfall, coloured pan, and/or Malaise traps. Berlese extractions of the substrate and/or active netting occurred at some sites. Both adults and larvae of aquatic species were collected using dip nets and bottle traps from a variety of freshwater habitat types: coastal rock bluff pools, tundra ponds, fen ponds, streams, lakes, and the Churchill River. Sampling was concentrated in areas accessible by road in Churchill, bounded by the Churchill River in the west, Hudson Bay in the north, a boundary approximating 58.6°N in the south, and a boundary approximating 93.4°W in the east. A small number of specimens were also collected at 11 sites outside these boundaries, including one site at Bylot Station (58.42°N, 94.13°W) and 10 sites in Wapusk National Park, including five sites near Nestor 1 field camp (58.66°N, 93.19°W), four sites near the mouth of the Broad River (58.42°N, 92.87°W), and one isolated relict beach ridge (57.58°N, 93.87°W).

All specimens were either pinned and air dried, or preserved in 95% ethanol. Specimens from earlier years were held at room temperature following collection, but aquatic specimens collected in 2010 were held in ethanol and at −20°C following the field season. Specimens are deposited at the Biodiversity Institute of Ontario, University of Guelph, Guelph, Ontario, Canada (BIOUG); the Wallis-Roughley Museum of Entomology, University of Manitoba, Winnipeg, Manitoba, Canada (WRME); and the Canadian National Collection of Insects, Arachnids and Nematodes, Ottawa, Ontario, Canada (CNC). Locality data, specimen photographs, voucher numbers, and identifier for all specimens are available through the Barcode of Life Data Systems (BOLD; http://www.boldsystems.org) [23] under project names: Coleoptera of Churchill [COCHU] and Aquatic Coleoptera of Churchill 2010 [EBCCH]. All specimens analyzed together for this study can be accessed as a single BOLD dataset dx.doi.org/10.5883/DS-COLCHU. Also see Additional file 1 for a list of specimens, taxonomy as of October 22, 2013, and locality data.

Generally, from each site, approximately 2–5 individuals per gross morphospecies within each family were selected for DNA barcoding. For a detailed freshwater study in 2010 including both adults and larvae, at least 10 individuals per morphospecies across sites were selected by EB, whenever available. Additional specimens were selected from groups that were difficult to distinguish or which possessed many individuals in larval form (e.g. the genus *Hydroporus* and family Gyrinidae). For some terrestrial and aquatic families, some of the selected individuals were identified to species level on the basis of morphology by a number of experts, with the identifier listed in the specimen record for each individual specimen on BOLD. Other individuals were identified to species level on the basis of genetic matches (see below) to other specimens in our dataset or to other records on BOLD. These are designated with “Digital Photograph; BOLD Barcode Library (Date)” in the Identification Method column; these cases were verified to be reasonable matches using the photographs. For families lacking available taxonomic expertise, specimens were identified to family or genus level by TW or EB [24,25], adjusted to the taxonomic hierarchy of Bouchard *et al.*[26], and a provisional interim species name was assigned based upon the DNA barcode results (see below) to enable provisional determination of species richness. Molecular Operational Taxonomic Unit (MOTU) names were assigned using sequential codes within genera, starting at CHU1 for Churchill. For MOTUs lacking genus-level identifications, a placeholder genus name (family name plus the initials TW) was inserted into the genus field in BOLD, to permit the distance analysis calculations to be performed on the MOTUs. Similarly, single morphospecies containing two or more provisional species based on the barcode results were assigned CHU codes onto the end of the binomial Linnaean name, with barcode clusters treated as separate species for richness analysis. This dataset and the associated records in BOLD will continue to gain species-level identifications over time; all identifications and interim names as analyzed in this paper are available in Additional file 1.

### DNA barcoding and alignment

Tissue samples consisting of one leg (occasionally two or more for small-bodied species) were removed from specimens and deposited into 96-well plates prefilled with 30 μl of 95% ethanol. All instruments used to remove leg tissues were cleaned in 95% ethanol and sterilized by flame between specimens. Most plates were submitted to the Canadian Centre for DNA Barcoding (CCDB) where DNA was extracted from tissue samples following standard invertebrate protocols [27,28]. Molecular processing of the 2010 aquatic samples (919 specimens) was performed using the manual version of the same protocols. The barcode region of cytochrome *c* oxidase subunit I (COI) was amplified using a polymerase chain reaction (PCR). Typically, most specimens were subjected to a second attempt at PCR amplification if the first attempt did not yield a full-length barcode sequence of at least 500 base pairs (bp) (see Additional file 2 for primer details). PCR amplification conditions, product checking, PCR cycle sequencing, and bidirectional sequencing followed standard protocols employed at the CCDB [29-31]. Forward and reverse sequences for each specimen were combined into a single consensus sequence and aligned using the CodonCode Aligner software v. 3.0.2 (CodonCode Corporation). Sequences and alignments were verified to be free of gaps and stop codons using the amino acid translation. The alignment was uploaded to BOLD and also imported into MEGA version 5.0 [32] for data analysis. Sequences, PCR and sequencing primers, and trace files are available for each individual specimen through the two projects on BOLD listed above (primer sequences and references in Additional file 2).

Sequences were verified as belonging to Coleoptera using Genbank BLAST and using data scrutiny tools available through BOLD (BIN discordance report, ID engine, photographic data, and NJ trees). Only high-quality sequences with a minimum length of 500 bp and containing fewer than 1% missing nucleotides (Ns) were retained for analysis of genetic divergence patterns to reduce intraspecific variations due to sequence length variation [23]. Sequences of at least 300 bp length were retained for providing provisional species identifications, for analyzing habitat occupancy, and for estimation of total biodiversity. Shorter sequences still provide reliable matches to conspecifics [33] and for biodiversity analysis it is beneficial to include the maximum data available.

### Data analysis

To visualize the barcode data, a neighbour-joining (NJ) tree [34] was built in MEGA 5.0 [32] using the following parameters: Kimura 2-Parameter (K2P) distance model [35] with pairwise deletion of gaps/missing data and inclusion of all substitutions (transitions and transversions). Although p-distances have recently been advocated for DNA barcoding studies, at low taxonomic levels K2P distances tend to be similar to p-distances [36,37]; thus, we use the more commonly applied former metric to facilitate comparison with other works. Sequences of at least 300 bp length were included in the NJ tree, but eleven short sequences were omitted because of lack of overlapping regions with other short sequences. These sequences were selected so as to minimize the total number of sequences omitted while also ensuring that no species was entirely deleted. A NJ analysis including all families was first performed to verify separation of the individuals morphologically assigned to different families. In order to facilitate bootstrap analysis (1000 replicates), NJ phenograms were subsequently constructed on four data partitions separately: the three largest families individually (Carabidae, Dytiscidae, Staphylinidae) and all remaining families together (see Additional file 3).

For families lacking species-level identifications, and when single morphospecies formed two or more clusters, we inspected the genetic distance matrices obtained through MEGA in order to assign provisional species codes (Additional file 4). Our Molecular Operational Taxonomic Units (MOTUs) were firstly defined based upon Barcode Index Numbers (BINs) [38], accessed through BOLD on Nov 21, 2012. BINs are genetic groupings assigned by BOLD3 for sequences that are least 500 bp in length. The BIN algorithm is based on a 2.2% initial seed sequence divergence that is combined with an algorithm that permits higher or lower divergences within BINs on the basis of genetic distance patterns [38]. We assigned our shorter sequences to these MOTUs if they clustered within a particular BIN, and we separated clusters lacking BINs (due to sequences being <500 bp) when they showed an average divergence of 3% or greater. Although threshold-based methods can be problematic for large spatial scales [22] and with comprehensive global taxonomic sampling [39], the early-proposed 2% threshold to separate intraspecific and interspecific divergences [40] is effective at discriminating most insect species within the Churchill region e.g. [18,19]. This value is slightly conservative in some insects, in that it underestimates the number of species in Diptera, which are hyper-diverse in Churchill [41]. We conservatively employed a 3% threshold, as short sequences may lie in more variable regions of the barcode sequence compared to the average for the entire barcode region. Therefore, our MOTU richness estimates are expected to represent minimum estimates of species-level richness. Closely related species that were morphologically identified yet show low genetic divergence were retained as separate species. While these may, in fact, represent single species with higher levels of morphological variation than known previously, the conservative approach dictates that there is insufficient evidence to overrule expert morphological identification.

Using the ≥500 bp data set, the maximum genetic distance among individuals within provisional species was calculated for all species having a sample size of at least two. Nearest interspecific distances were calculated for each species, as being the genetic distance to the nearest neighbouring sequence of a different species. These distances, both obtained using the Barcode Gap Analysis function in BOLD3, were then plotted on a histogram using R [42].

Based on recorded field data, each specimen was assigned to one of 22 habitat classifications, which covered both aquatic and terrestrial habitats (Additional file 5). The completeness of our survey was investigated by constructing randomized accumulation curves of provisional species for: all species together, species belonging to the three most dominant families separately (Carabidae, Dytiscidae, Staphylinidae), and for four dominant habitat types separately. The habitats include Dry Forest, Wet Forest, Open Wet, and Open Dry habitat (i.e. dry tundra, thinly vegetated or unvegetated gravel or sand). Wet habitats include both aquatic habitats (ponds, lakes, streams) and wet terrestrial and semi-terrestrial habitats such as riparian zones, fens, and bogs. All other habitat types were designated as dry. Forested habitats were those for which significant tree cover was reported, and included ponds and riparian zones in forested areas (Forested Wet). Species accumulation curves were built with 1000 permutations and sub-sampling without replacement [43] in the R package Vegan [42,44]. The Chao1 [45] biodiversity estimator was calculated for the entire collection (all 3203 individuals with sequences of ≥300 bp) using EstimateS 8.2 [46].

Geographic ranges of Carabidae and Dytiscidae species, two of the most speciose families in this study, were reviewed [2-4,7,12,47,48] to investigate possible sources of colonization (Beringia, southern open ground, or forested habitats) that could provide insight into present distribution patterns in the Churchill region. It is expected that colonization in open-ground habitats proceeded from Beringia or Arctic coastal refugia (leading to modern distributions west of Hudson Bay and north of the tree line) and from the south following glacial retreat (leading to modern distributions that include barren ground parts of Quebec and Labrador). Forest dwellers would be expected to have modern transcontinental distributions [7,49]. For each named carabid and dytiscid species with at least five specimens, a crosstab (chi-square) analysis was performed (PASW Statistics 19, SPSS, Inc., 2010, Chicago, IL, http://www.spss.com). These analyses test for differences in distribution of individuals among the four dominant habitat categories from what would be expected if the specimens were randomly distributed, based on total specimens collected from each habitat as an approximation of sampling effort.

## Results

Barcodes of at least 300 bp in length were obtained successfully for 3203 specimens, representing 302 total species and provisional species in 31 families of Coleoptera (Table 1). 2972 (93%) of these successful sequences were ≥500 bp in length. As 3803 specimens were originally selected for barcode analysis, the overall barcoding success rate was therefore 84.2% at the 300 bp sequence length cut-off and 78.1% at 500 bp. Barcoding success rates improved over time, which may have been due to improved laboratory and preservation methods. For example, the 2010 aquatic samples (specimens in BOLD project EBCCH) were preserved in ethanol, which was exchanged following initial preservation, and also held at −20°C immediately after the field season; the success rates for this subset were 94.3% at ≥300 bp and 93.9% at ≥500 bp.

**Table 1:** Summary of specimens included in the Churchill Coleoptera barcode library

**Family**	Number of genera*	**Provisional species richness**	**Number of named species**	**Number of individual barcodes (≥300 bp)**
		**(named species plus MOTUs)**		
Bostrichidae**	0	0	0	0
Brachyceridae	1	1	0	1
Buprestidae	3	3	3	4
Byrrhidae	2	6	3	12
Cantharidae	2	6	0	36
Carabidae	21	52	46	778
Cerambycidae	5	5	5	12
Chrysomelidae	9	10	4	198
Cleridae	0	1	0	1
Coccinellidae	8	12	6	63
Cryptophagidae	2	7	0	49
Cucujidae	1	1	1	1
Curculionidae	10	15	10	46
Dytiscidae	17	63	52	1338
Elateridae	6	10	8	58
Elmidae	1	1	1	1
Gyrinidae	1	9	7	124
Haliplidae	1	5	3	44
Heteroceridae	1	1	1	5
Hydraenidae**	0	0	0	0
Hydrophilidae	5	11	8	84
Lampyridae	1	1	1	13
Latridiidae	2	2	0	6
Leiodidae	3	10	2	39
Melyridae	0	2	0	5
Mordellidae	0	2	0	2
Ptiliidae	0	2	0	3
Scarabaeidae	2	2	2	4
Scirtidae	1	3	2	72
Scraptiidae	0	1	0	4
Silphidae	2	3	3	35
Sphindidae	0	1	0	6
Staphylinidae	22	54	16	159
**Total**	**129**	**302**	**184**	**3203**

*Minimum number, as 123 specimens were identified to family level only.

**Family collected in Churchill but failed to yield a successful barcode sequence.

**Figure 2:**
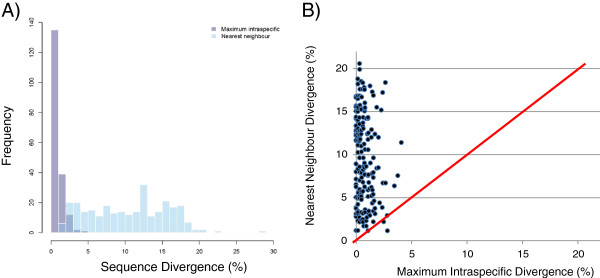
**Maximum intraspecific and nearest neighbour (interspecific) divergences for all species of Coleoptera in the Churchill barcode library, based upon barcode sequences of at least 500 bp.** A histogram **(A)** indicates overlap in the ranges of these values, while a plot with each species represented as a point **(B)** shows that nearly all species fall above the red 1:1 line, reflecting genetic separation between conspecifics and neighbouring species.

Of the 184 species in our dataset that were identified to the level of a binomial (Linnaean) species name, all but four (97.8%) of these fell into one or more separate MOTUs using the BIN definition, which displayed more than 2% sequence divergence (sequences of ≥500 bp) from all other species (Figure 2). The exceptions involved two pairs of closely related species: *Agabus phaeopterus* and *A. thomsoni* (family Dytiscidae), which showed a nearest neighbouring sequence distance of 1.15%, and *Amara alpina* and *A. torrida* (Carabidae), which showed a minimum of 1.19% divergence. In both cases, the sister species pair shares a single BIN and also displays reciprocal monophyly, but in both cases with low bootstrap support (<50%) for one of the two species in the pair (Additional file 3). Of the remaining species, 170 (92.4% of the total of 184) formed a single MOTU, while 10 species (5.4% of the total) (*Agabus antennatus*, *A. bifarius, Cymindis unicolor, Elleschus ephippiatus, Gyrinus dubius, Hydrobius fuscipes, Hygrotus novemlineatus, Philonthus boreas, Sericus incongruus*, *Simplocaria metallica*) were separated into two MOTUs. Six of these pairs of MOTUs were reciprocally monophyletic, while two were more widely separated by relatives on the tree (*A. antennatus*, *H. novemlineatus*). The two MOTUs (BINs) of *C. unicolor* formed a paraphyletic/monophyletic relationship in some NJ reconstructions (“other families” bootstrap analysis; Additional file 3) but a reciprocally monophyletic relationship in others (all-specimen analysis; not shown), indicating an uncertain relationship between these MOTUs based upon the analysis presented here. There were 108 MOTUs that were genetically distinct from all others but lacked species-level identifications at the time of publication. Species names may further be filled in for these records on BOLD over time as taxonomic expertise becomes available or new species are described. These MOTUs were treated as provisional species for further biodiversity analysis.

The maximum K2P divergences within species (including provisional species) ranged from 0–4.1% (average of the maxima of 0.75%), based upon a sample size of 189 species that were represented by 2 or more sequences of at least 500 bp. Minimum interspecific distances (distances to the single nearest neighbour sequence belonging to a different species) ranged from 1.15-28.1% (mean of 10.4%) (Additional file 4), based upon a sample size of 283 species with at least 1 sequence of ≥500 bp. The NJ phenograms (Additional file 3) provide a visualization of the barcode distances in this dataset.

**Figure 3:**
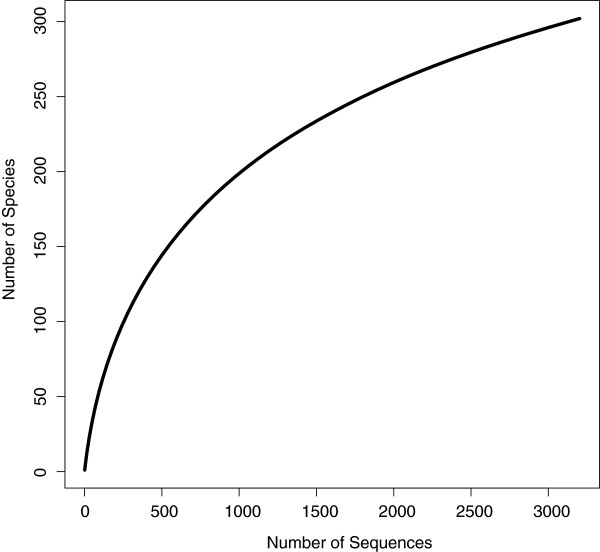
**Accumulation curve for all species of Coleoptera in the Churchill barcode library.** This individual-based rarefaction curve included all 3203 specimens with sequences of ≥300 bp and was based upon 1000 permutations.

**Figure 4:**
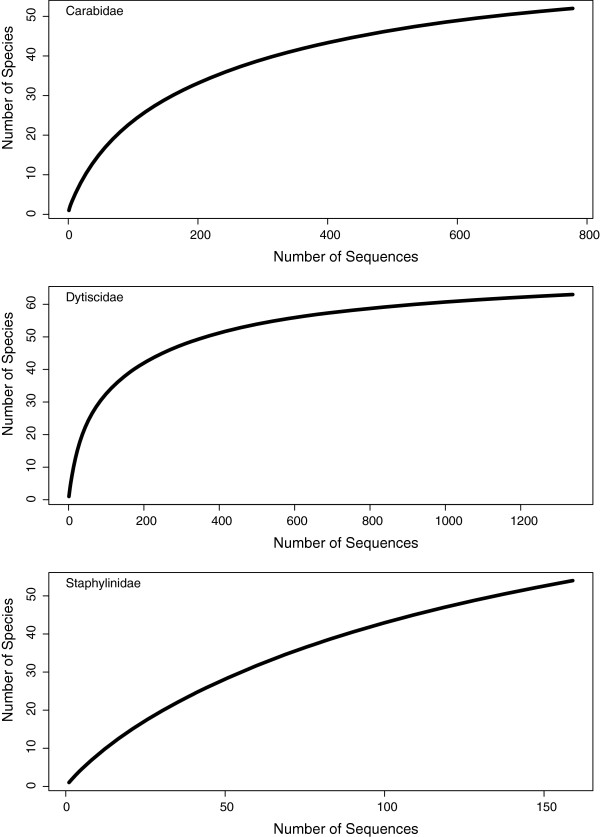
Accumulation curves for major families of Coleoptera in the Churchill barcode library.

**Figure 5:**
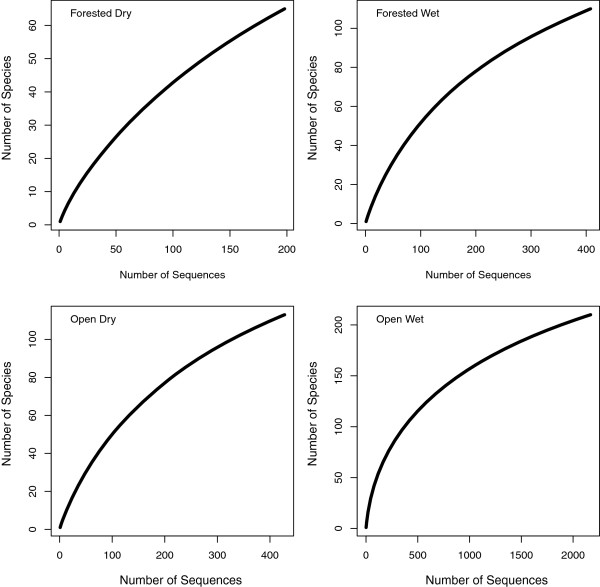
Accumulation curves by broad habitat category.

The total species accumulation curve indicates that there is a large number of additional species remaining to be sampled in the Coleoptera of the region (Figure 3). Of the 302 total species and provisional species, 92 are represented by a single individual (singletons), while 44 are doubletons. The mean sample size per species is 10.6, while the median is 3. The Chao1 biodiversity estimator indicated there are likely to be approximately 395 species (95% C.I. 357–460) of Coleoptera present in this region. Examination of accumulation curves of the dominant families indicates that much of the undocumented diversity is within the Staphylinidae, and that Carabidae and Dytiscidae are well-sampled (Figure 4). When separated by broad habitat category, only the Open Wet habitat accumulation curve suggests adequate sampling (Figure 5), which reflects the focus on aquatic beetles, particularly dytiscids, in the collecting. The other three habitat categories indicate a considerable number of species remains to be sampled, and Dry Forest was particularly undersampled.

## Discussion

### Diversity and familial composition of Coleoptera of Churchill

Biodiversity of Coleoptera shows a strong tendency to decline at northern latitudes. Although our study led to a substantial increase in the known fauna from Churchill, from ca. 60 to >300 species and provisional species, and from 20 to 33 families, our results based upon six years of collecting and DNA barcoding of >3000 specimens nevertheless confirm the relatively impoverished fauna compared with that of more southerly locations [2,5-7]. Moreover, there is a relative paucity of phytophagous and soil-dwelling detritivorous species. Predatory families such as Carabidae, Dytiscidae, and Staphylinidae are the most abundant and diverse groups in both terrestrial and aquatic habitats, and accumulation curves suggest that the latter is even more diverse than reported here. Danks [3] suggested that northern ecosystems are more strongly regulated by abiotic conditions, such that resources such as plant litter and living plant tissue are incompletely exploited. Moreover, the diversity of food sources for phytophages also decreases with latitude [50]. Phytophagous and saprophagous Coleoptera, represented in this study by the families Chrysomelidae, Elateridae, Cerambycidae, Curculionidae, Cryptophagidae, and Leiodidae, may be largely unable to adapt to the soil and climatic conditions as well as lower plant diversity [51] and are thus proportionally less represented in Churchill in comparison with their overall Canadian diversity [2,7].

In the analysis of carabid and dytiscid habitat distributions, approximately 76% of the specimens in these families were collected in Open Wet habitats, particularly bluff and tundra pools. Dytiscids were, as expected, almost completely absent from dry sites, while carabids in wet habitats were not collected from truly aquatic, submerged habitats. Among named species with five or more records, only two species of Dytiscidae (*Ilybius erichsoni*, *Neoscutopterus hornii*) showed a significant preference for Forested Wet habitats, which is in agreement with published modern distributions [52]. Thirteen species were found most frequently in Open Wet habitats (Table 2). The remaining 22 named species showed no significant deviation from random, suggesting that aquatic habitats in both forested and open areas were suitable. Within the Carabidae, only *Notiophilus aquaticus* showed a significant bias toward Forested Wet habitats, which is in agreement with published modern distributions [7,53]. *Elaphrus americanus* preferred Open Wet habitats, and *Pterostichus caribou* preferred both Open Wet and Open Dry habitats. Of the remaining species with five or more records, eight species showed a significant preference for Open Dry habitats, while nine species showed no preference.

**Table 2:** Summary of crosstab test results for broad habitat distributions (open vs. forested, hygrophilous/aquatic vs. dry) of carabid and dytiscid beetle species in the Churchill region

**Species**	Distribution*	Habitat preference**	**n**	**p-value**	**Distributional references**
**Carabidae**					
*Amara alpina*	A	Open, dry	19	0.024	[7,12,48,53]
*Amara quenseli*	SA	Open, dry	29	0.021	[48,53]
*Amara sinuosa*	S	-	8		
*Bembidion hastii*	SA	-	5		[7,47,48,53]
*Bembidion sordidum*	WC	-	5		[7,48,53]
*Calathus ingratus*	SLA	-	11		[53]
*Carabus chamissonis*	SHA	Open, dry	14	0.046	[7,47,48,53]
*Carabus maender*	SLA	-	11		[7,47,48,53]
*Carabus taedatus agassii*	SAxB	Open, dry	450	<0.01	[47,48,53]
*Cymindis unicolor*	WC	-	15		[7,12,53]
*Dyschirius hiemalis*	SA	-	5		[54]
*Elaphrus americanus americanus*	SA	Open, wet	23	<0.01	[6,53]
*Elaphrus lapponicus*	SLA	-	7		[7,48,53]
*Notiophilus aquaticus*	SHA	Forested, wet	9	0.02	[7,53]
*Notiophilus borealis*	WC	Open, dry	6	0.05	[7,53]
*Pterostichus brevicornis brevicornis*	SHA	Open, dry	16	0.01	[7,53]
*Pterostichus caribou*	A	Open	10	0.05	[7,8,12,53]
*Pterostichus pinguedineus*	SHA	-	5		[7,12,48,53]
*Pterostichus punctatissimus*	SLA	Open, dry	28	0.012	[7,12,53]
*Stereocerus haematopus*	WC	Open, dry	31	<0.01	[7,48]
**Dytiscidae**					
*Agabus ajax*	WC	Open	51	<0.01	[52]
*Agabus antennatus*	WC	Open	35	<0.01	[52]
*Agabus arcticus*	SLA	-	52		[49,52]
*Agabus audeni*	WC	-	9		[52]
*Agabus bicolor*	WC	Open	10	<0.01	[52]
*Agabus clavicornis*	SHA	-	9		[52]
*Agabus colymbus*	SHA	Open	32	<0.01	[52]
*Agabus infuscatus*	S	Open	93	<0.01	[7,52]
*Agabus phaeopterus*	S	-	22		[52]
*Agabus thomsoni*	S	-	8		[52]
*Carrhydrus crassipes*	WC	-	7		[52]
*Colymbetes dolabratus*	SA	-	180		[7,52]
*Dytiscus alaskanus*	WC	Open	9	<0.01	[52]
*Dytiscus dauricus*	SA	-	36		[52]
*Graphoderus perplexus*	SA	-	16		[52]
*Hydroporus dentellus*	SA	-	9		[52]
*Hydroporus erythrocephalus*	?	Open	11	<0.01	
*Hydroporus fuscipennis*	SA	Open	5	<0.01	[52]
*Hydroporus morio*	SHA	Open	101	<0.01	[52]
*Hydroporus sinuatipes*	SLA	Open	24	0.03	[52]
*Hydroporus striola*	SLA	-	6		[52]
*Hydroporus tenebrosus*	SA	-	12		[52]
*Hygrotus marklini*	SA	-	16		[52]
*Hygrotus novemlineatus*	A	Open	39	<0.01	[52]
*Hygrotus picatus*	SA	-	5		[52]
*Hygrotus sayi*	S	-	6		[52]
*Hygrotus unguicularis*	WC	Open	57	<0.01	[52]
*Ilybius discedens*	SA	-	6		[52]
*Ilybius erichsoni*	S	Forested	14	0.05	[52]
*Ilybius subaeneus*	S	-	44		[52]
*Laccophilus biguttatus*	SA	-	25		[52]
*Nebrioporus macronychus*	?	Open	24	<0.01	
*Neoscutoperus hornii*	SLA	Forested	5	0.05	[52]
*Oreodytes davisii*	?	-	20		
*Rhantus gutticollis*	S	-	5		
*Rhantus wallisi*	SA	-	39		[52]
*Stictotarsus griseostriatus*	SA	-	96		[52]

Only those species with at least five specimens collected are considered. Canadian provincial records summarized in Bousquet et al. [2]; other available distributional references are supplied below.

*distributional classes from Danks [7] A = Arctic, southern boundary north of the tree line; SA = southern Arctic, transcontinental north of the tree line; SAxB = southern Arctic excluding Beringia, transcontinental north of the tree line; WC = western and central, range from western North America east to Hudson Bay; S = southern, northern boundary at the tree line; SHA = southern high arctic, arctic species extending south of the tree line; SLA = southern low arctic, southern species extending north of the tree line.

** all Dytiscidae were collected in aquatic habitats.

A steep latitudinal diversity gradient is seen in the Lepidoptera [deWaard et al. unpubl.], although both the Hymenoptera [21,55] and Diptera [41], Wang et al. unpubl.] are highly speciose in Churchill. However, for these latter two groups, it is currently challenging to assess the strength of their latitudinal diversity gradient due to the lack of studies in multiple regions employing genetic methods. DNA barcoding has often revealed higher diversity than traditional taxonomy, particularly in parasitoid groups [55-58]. Nevertheless, the striking diversity of Hymenoptera and Diptera in the north suggests that these groups may have radiated into some of the niches occupied by beetles at more southerly latitudes. Intriguingly, the Hymenoptera fauna of Churchill is highly dominated by parasitoid species [21], which may mirror our detected pattern of a greater proportional representation of predator beetle families.

### Geographic origin of the Coleoptera fauna of Churchill

The current insect assemblage in the Churchill region is relatively young, incorporating species from several different refugia that colonized following the LGM, most notably species from Beringia, which followed the deglaciation of the Arctic coast and then south to Churchill, and species from the south that followed the ice margins northward. Dispersal ability of insects and their colonization patterns following the LGM may provide information on their potential responses to current climate warming [13]. The ongoing warming of the Churchill River resulting from damming and climate change may enhance its role as a corridor for northwards dispersal of both the terrestrial and aquatic beetles to the region [13]. Additional sampling of beetles in some habitats, particularly dry boreal forest, as well as further barcoding of beetles from other regions of Canada are both necessary to complete a phylogeographic analysis of likely species colonization routes following the LGM. Spitzer and Danks [59] also suggest that boreal forest peatlands, with their high habitat diversity and potential as refugia and habitat islands, could harbour endemic and relict species and contain significant and undocumented richness.

Danks [3] notes that 51% of boreal insect species have a transcontinental distribution, but only 4% of tundra/open ground species do. Other sources, however, indicate that many species have distributions that include habitats north of the tree line east of Hudson Bay in Quebec, Labrador, and sometimes Newfoundland, suggesting that retreating ice was followed by recolonization from the south, rather than assuming dispersal across Hudson Bay [2,52]. Danks [3] also points out that warming occurred prior to significant retreat of the ice margins, and many species persisting in southern refugia may not have survived that increase in temperature. Ten of the 13 dytiscid species that we found to have a preference for aquatic habitats in Open areas (Table 2) are reported as occurring only west of Hudson Bay by Bousquet et al. [2], suggesting the difficulty of dispersal across Hudson Bay, although Larson [52] records many dytiscid species as transcontinental. Exceptions to this pattern are *Hydroporus fuscipennis*, which occurs across Canada in boreal and grassland lakes and ponds, and *H. notabilis*, which occurs in the low arctic across Canada, preferring peat pools [2,52].

Distribution patterns of Carabidae species are more complex. Of the 11 species that showed a significant habitat preference (Table 2), nine prefer Open Dry habitat. Most of these species are currently distributed north of the tree line on open tundra [2,12,47,53,60], with fossil records from areas in Yukon, Alaska, and other regions that had a similar dry tundra habitat during the LGM [48,61]. *Carabus taedatus* is a notable exception, with a fossil record from the Northwest Territories [48] but not Beringia [53]. *Amara quenseli* may be found throughout Canada, typically in open grassy habitat [2,53], with a fossil record in Siberia that suggests a Beringian origin [48]. *Notiophilus aquaticus*, with a preference for Forested Wet habitat in this study, is circumpolar in distribution and found throughout Canada [2,47,53]. It is reported by Lindroth [47] as occurring on open ground, although Danks [7] indicates that it is found in both forested and tundra habitats. Garry [10] documented 65 species of Carabidae northwest of Churchill in the drainages of the Caribou and Seal Rivers (53 woodland, 34 tundra, 22 common to both major habitat types). Many species are well represented in midwestern fossil assemblages and possibly followed the retreating glaciers in appropriate habitats, in contrast with Danks’s [3] view that many Churchill species represent Beringian fauna. Garry [10] notes, however, that for at least 25 of the species he recovered, the glacial refugium and route(s) of recolonization are not clear.

*Elaphrus americanus*, with a preference for Open Wet habitat in this study, occurs throughout much of North America in moist habitats [2,47,53]. During the LGM it persisted in both Beringia and south of the ice sheet, with some morphological divergence of these refugial lineages observed [6,62]. Our results show a maximum intraspecific divergence of 1.4% in this species, which could indicate that multiple refugial lineages may be present in Churchill. Other named species with sufficient intraspecific divergence to suggest multiple refugial lineages or multiple species include *Simplocaria metallica* (Byrrhidae), *Cymindis unicolor* (Carabidae), *Elleschus ephippiatus* (Curculionidae), *Agabus antennatus*, *A. bifarius*, *Hygrotus novemlineatus* (Dytiscidae), *Sericus incongruus* (Elateridae), *Gyrinus dubius* (Gyrinidae), *Hydrobius fuscipes* (Hydrophilidae), and *Philonthus boreas* (Staphylinidae).

### DNA barcoding of Coleoptera

Globally, Coleoptera are the largest order of insects in terms of described species diversity [1]. Despite "the Creator’s inordinate fondness for beetles" [63], Coleoptera have not been favoured to date by barcoders. For example, using the public data portal available through BOLD3 (accessed June 24, 2013), there were ca. 82 K public barcode records for beetles representing ca. 19 K provisional species. By comparison, the others of the top four most diverse insect orders are represented by approximately 2.5-fold (Hymenoptera), 3.5-fold (Diptera), and 8.5-fold (Lepidoptera) more public records. Moreover, several important studies on genetic variability within and between Coleoptera species have largely employed genetic regions other than the standard animal barcode region e.g. [22,64]. Thus, the DNA barcoding of Coleoptera is in its infancy, especially when considering their described [1] and projected [65] global diversity.

Although our study contributes important regional-scale data (and ~4% of the total) to the pool of barcode data for Coleoptera, further sampling from other regions is required to be able to use these data to “test” DNA barcoding for this taxon. Using a different region of the COI gene, Bergsten et al. [22] determined that the success rate for identifying individuals of a group of aquatic beetles (tribe Agabini, family Dytiscidae) to morphological species was nearly 100% at small spatial scales. However, for this taxon, this diminished to ~90% at continental scales, or lower, depending upon the match criteria employed. Further assessment across numerous beetle families will be required to further evaluate how well COI variability delineates species boundaries in the Coleoptera at broad spatial scales. Additionally, integrative methods incorporating genetic, morphological, and ecological information e.g. [56-58] may be needed, rather than assuming that morphospecies represent *true* species boundaries against which to test DNA barcodes. Our data are useful for such future tests, as the locality and sequence data are available online, and vouchers are retained for all barcoded specimens and are housed in publically accessible collections.

Being confined to the Churchill region, our study was originally expected to be a zone of a large “barcoding gap” [39], in which there is a discontinuity between intraspecific vs. interspecific divergences for two main reasons. First, the total richness of Coleoptera species is less than that in more southerly regions. Second, there may have been pruning of intraspecific genetic variation by glaciations [66,67]. Our results, which are based upon a mixture of named species and provisional species, indeed do support a general difference in intraspecific vs. interspecific divergences for most species among the Churchill beetles as well as a projected high rate of success in future barcode-based identifications for beetles of this region. Of the 184 named species, only 4 (2.2%) displayed sharing of MOTUs under the BIN definition, and all others were readily separated into one or more MOTUs. However, we observed major overlap in the total divergence ranges (maximum intraspecific up to 4.1% and minimum interspecific down to 1.1%). Thus, this high rate of success at separating species reflects the general effectiveness of the BIN algorithm in recovering clusters/species of Churchill beetles. Meanwhile, new specimens belonging to the two pairs of closely related species could still be identified on the basis of their barcodes, since both members of these species pairs are now in the data set and they do show some genetic differentiation. Interestingly, the family Muscidae (Diptera) similarly does not show a clear barcoding gap [41], despite the extremely high effectiveness of barcode-based species-level identification in the muscids using clustering methods. Despite a lack of a universal barcoding threshold, the strong species-level clustering pattern observed here and in other Churchill invertebrates points towards success for future barcode-based studies of beetles in the Churchill region and other northern regions. Well-populated datasets do not require a strict threshold to hold true for identification success to be achieved [39,41]. Thus, near-comprehensive surveys, such as being conducted for the Churchill region, are a valuable resource for the further study of biodiversity.

## Conclusions

The barcode library of Coleoptera presented here represents a valuable tool for the evaluation of long-term change in northern Canada. Although insects are generally more mobile in response to changing conditions than vegetation, many species demonstrate affinity to particular vegetation assemblages, and northern movement of the "tree line" could have significant effects on the fauna of the Churchill region. These slower changes in vegetation distribution, particularly at an ecological boundary such as the tree line in the Churchill region, could limit the availability of suitable habitats north of the current range limits for some species, slowing their ability to move northward despite increased warming rates and diminishing availability of suitable habitat in the south [13]. Furthermore, the loss of genetic diversity in populations of both open ground and boreal forest species could seriously affect their ability to adapt to both present and future change. Open-ground species could be negatively affected by the combination of founder bottlenecks and retreat of the southern boundary of their distributions, even as suitable habitat expands to the north [13,68,69]. Similarly, changes at the southern edge of the distribution of forest-dwelling species may lead to fragmentation of the habitat and coincident loss of overall genetic diversity in the biogeographic center of distribution. Climate and habitat changes following the LGM proceeded much more slowly than are being observed today, enabling dispersal from refugia across more constant biotic (vegetation) and abiotic (climate) habitat conditions [13,70]. Our study is linked to a taxonomically broader “Barcoding Biotas” survey of the Churchill region, which enables co-ordinated monitoring of the influences of climate change and invasive species across taxonomic groups in this model boreal/sub-arctic transition zone.

## Competing interests

The authors declare that they have no competing interests, financial or otherwise.

## Authors’ contributions

TSW, EB, and SJA designed the study; TSW, EB, RER, PGK, and DS conducted and co-ordinated the field work; TSW, EB, ABTS, RER, and HG identified most of the specimens; EB performed molecular laboratory analysis of the 2010 aquatic specimens; SJA, DS, and EB managed and validated the molecular data; TSW, RNL, EB, and SJA analyzed the data; TSW and EB prepared the figures; TSW drafted most sections of the manuscript; SJA and EB drafted sections of the manuscript; and all authors commented on the manuscript and approved the final version.

## Authors’ information

TSW is a Research Associate with the Canadian Pollination Initiative (NSERC-CANPOLIN) and Adjunct Professor in the School of Environmental Sciences at the University of Guelph (UG). EEB is a recent graduate of the Masters program in the Department of Integrative Biology (UG) and is interested in understanding the phylogenetic patterns of freshwater insect communities. RER was Professor of Entomology and curator of the J.B. Wallis Insect Museum (now the Wallis-Roughley Insect Museum) at the University of Manitoba. PGK is Professor Emeritus in the School of Environmental Sciences at the University of Guelph and Scientific Director of the Canadian Pollination Initiative strategic network. RNL is a collections technician at the Biodiversity Institute of Ontario (UG). ABTS is a Research Associate of the Canadian Museum of Nature and provided and coordinated Coleoptera identifications for specimens represented in BOLD. HG is recently retired from the Canadian National Collection of Insects, Arachnids and Nematodes, where he investigated the systematics, ecology, morphology, diversity and faunistics of sawflies and ground beetles. DS is a Research Associate at the Biodiversity Institute of Ontario (UG) and is interested in evolutionary ecology, speciation processes, and phylogenomics. SJA is an Assistant Professor in the Biodiversity Institute of Ontario & Department of Integrative Biology (UG) and is interested in evolutionary community structure, macroevolution, and using genetic tools to elucidate biodiversity.

This paper is dedicated to the memory of Dr. Robert Roughley, who died in November 2009. Dr. Roughley spent his career studying the systematics and biology of Coleoptera, particularly the Dytiscidae and the beetle fauna of Manitoba.

## Supplementary Material

Additional file 1 Complete collection data for all individual specimens. Click here for file

Additional file 2 **List of primers.** Forward (F) and reverse (R) primers typically used to amplify COI sequences of Coleoptera of Churchill, although other primers were tried on a trial basis for a small number of specimens. The specific primers used for PCR and sequencing are available for all specimens through BOLD (http://www.boldsystems.org). Unless otherwise specified in footnotes, the listed primers are used for both PCR amplification and cycle sequencing. Typically, most specimens received two attempts at PCR with different primer sets, with the selection for first pass and second pass on the failures varying across years of the study. The C_LepFolF/C_LepFolR cocktail and LCO1490_t1/HCO2198_t1 have been found to be the most successful primer combinations for the Churchill beetles. Click here for file

Additional file 3 **Neighbour-joining trees of barcode sequences of Coleoptera of Churchill.** Neighbour-joining phenograms based upon Kimura-2-parameter genetic distances for 3194 COI sequences (≥300 bp) from Coleoptera specimens from Churchill. Clusters representing species or provisional species (see Methods) are collapsed into triangles, with the vertical dimension corresponding to sample size and the horizontal dimension corresponding to intraspecific genetic variability. Bootstrap values are based on 1000 pseudoreplicates, with values shown for nodes having values ≥70%. All sequences of at least 300 bp were included, except in cases where there was a lack of overlapping nucleotides among sequences (ProcessIDs of specimens omitted from analysis: TWCOL605-10, TWCOL141-09, TWCOL005-09, TWCOL204-09, AWWBC026-09, HMCOC345-07, TWCOL286-09, TWCOL080-09, TWCOL402-10, EBCCH402-1, and HMCOC696-09). To enable bootstrap analysis, 4 data partitions were run separately: A) family Carabidae; B) family Dytiscidae; C) family Staphylinidae; and D) all other families together. Click here for file

Additional file 4 **Genetic distances within and between 283 species or provisional species of Coleoptera of Churchill, based upon the 2972 specimens having barcode sequences of at least 500 bp.** “N/A” for the maximum intraspecific distance indicates a sample size of just one specimen of sequence length of at least 500 bp for that species. Additional species or provisional species only represented by sequences of <500 bp occur in the list of specimens (Additional file 1). Click here for file

Additional file 5 Summary of collection habitats for specimens with species determinations in the Churchill Coleoptera barcode library. Click here for file
