# Supplementary material for: The diversity and biogeography of the Coleoptera of Churchill: insights from DNA barcoding
Source: BMC Ecol. 2013 Oct 29;13:40. doi: 10.1186/1472-6785-13-40 (PMC3819705; doi:10.1186/1472-6785-13-40)
Supplement: Additional file 2 — List of primers. Forward (F) and reverse (R) primers typically used to amplify COI sequences of Coleoptera of Churchill, although other primers were tried on a trial basis for a small number of specimens. The specific primers used for PCR and sequencing are available for all specimens through BOLD (http://www.boldsystems.org). Unless otherwise specified in footnotes, the listed primers are used for both PCR amplification and cycle sequencing. Typically, most specimens received two attempts at PCR with different primer sets, with the selection for first pass and second pass on the failures varying across years of the study. The C_LepFolF/C_LepFolR cocktail and LCO1490_t1/HCO2198_t1 have been found to be the most successful primer combinations for the Churchill beetles. [file 1472-6785-13-40-S2.pdf]

Additional file 2 – List of primers

Forward (F) and reverse (R) primers typically used to amplify COI sequences of Coleoptera of Churchill, although other primers were tried on a trial basis for a small number of specimens. The specific primers used for PCR and sequencing are available for all specimens through BOLD (www.boldsystems.org). Unless otherwise specified in footnotes, the listed primers are used for both PCR amplification and cycle sequencing. Typically, most specimens received two attempts at PCR with different primer sets, with the selection for first pass and second pass on the failures varying across years of the study. The C_LepFolF/C_LepFolR cocktail and LCO1490_t1/HCO2198_t1 have been found to be the most successful primer combinations for the Churchill beetles.

Primer pair	Sequence (5' to 3')		References (F/R if different)
	F	R	
**Most common first-pass primers:**			
LCO1490/HCO2198	GGTCAACAAATCATAAAGATATTGG	TAAACTTCAGGGTGACCAAAAAATCA	1
LCO1490_t1/HCO2198_t1	TGTAAAACGACGGCCAGTGGTCAACAAATCATAAAGATATTGG	CAGGAAACAGCTATGACTAAACTTCAGGGTGACCAAAAAATCA	2,3[a]
LepF1/LepR1	ATTCAACCAATCATAAAGATATTGG	TAAACTTCTGGATGTCCAAAAAATCA	4
C_LepFolF/C_LepFolR	LepF1:LCO1490	LepR1:HCO2198	5
**Second pass primers (used on failures):**			
MLepF1 /HCO2198_t1	GCTTTCCCACGAATAAATAATA	CAGGAAACAGCTATGACTAAACTTCAGGGTGACCAAAAAATCA	6[b]/2

LCO1490_t1/MLepR1	TGTAAAACGACGGCCAGTGGTC AACAAATCATAAAGATATTGG	CCTGTTCCAGCTCCATTTTC	2/6[b]

[1] Folmer O, Black M, Hoeh W, Lutz R, Vrijenhoek R: **DNA primers for amplification of mitochondrial cytochrome c oxidase subunit I from diverse metazoan invertebrates.** *Mol Mar Biol Biotechnol* 1994, **3:**294-299.

[2] Floyd R. Published on BOLD. Folmer primers [1] tailed with M13 [3] sequences.

[3] Messing J: **New M13 vectors for cloning**. *Method Enzymol* 1983, **101**:29–71.

[4] Hebert PDN, Penton EH, Burns J, Janzen DH, Hallwachs W: **Ten species in one: DNA barcoding reveals cryptic species in neotropical skipper butterfly, *Astraptes fulgerator.*** *PNAS* 2004, **101:**14812-14817.

[5] Ivanova N. Published on BOLD. Cocktail is a mixture of primers [1,4].

[6] Hajibabaei M, Janzen S, Burns JM, Hallwachs W, Hebert PDN: **DNA barcodes distinguish species of tropical Lepidoptera.** *PNAS* 2006, **103:**968-971.

[a] These tailed Folmer sequences are used for PCR amplification, while the M13 tail sequences alone are used for cycle sequencing (M13F-TGTAAAACGACGGCCAGT; M13R-CAGGAAACAGCTATGAC).

[b] These "mini" primers [6] are used in combination with the Folmer [1] or other primers to generate two over-lapping fragments that can yield a full-length barcode sequence.
