# Supplementary material for: The diversity and biogeography of the Coleoptera of Churchill: insights from DNA barcoding
Source: BMC Ecol. 2013 Oct 29;13:40. doi: 10.1186/1472-6785-13-40 (PMC3819705; doi:10.1186/1472-6785-13-40)
Supplement: Additional file 3 — Neighbour-joining trees of barcode sequences of Coleoptera of Churchill. Neighbour-joining phenograms based upon Kimura-2-parameter genetic distances for 3194 COI sequences (≥300 bp) from Coleoptera specimens from Churchill. Clusters representing species or provisional species (see Methods) are collapsed into triangles, with the vertical dimension corresponding to sample size and the horizontal dimension corresponding to intraspecific genetic variability. Bootstrap values are based on 1000 pseudoreplicates, with values shown for nodes having values ≥70%. All sequences of at least 300 bp were included, except in cases where there was a lack of overlapping nucleotides among sequences (ProcessIDs of specimens omitted from analysis: TWCOL605-10, TWCOL141-09, TWCOL005-09, TWCOL204-09, AWWBC026-09, HMCOC345-07, TWCOL286-09, TWCOL080-09, TWCOL402-10, EBCCH402-1, and HMCOC696-09). To enable bootstrap analysis, 4 data partitions were run separately: A) family Carabidae; B) family Dytiscidae; C) family Staphylinidae; and D) all other families together. [file 1472-6785-13-40-S3.pdf]

Additional file 3 – Neighbour-joining trees of barcode sequences of Coleoptera of Churchill.

Neighbour-joining phenograms based upon Kimura-2-parameter genetic distances for 3194 COI sequences (≥300bp) from Coleoptera specimens from Churchill. Clusters representing species or provisional species (see Methods) are collapsed into triangles, with the vertical dimension corresponding to sample size and the horizontal dimension corresponding to intraspecific genetic variability. Bootstrap values are based on 1000 pseudoreplicates, with values shown for nodes having values ≥70%. All sequences of at least 300 bp were included, except in cases where there was a lack of overlapping nucleotides among sequences (ProcessIDs of specimens omitted from analysis: TWCOL605-10, TWCOL141-09, TWCOL005-09, TWCOL204-09, AWWBC026-09, HMCOC345-07, TWCOL286-09, TWCOL080-09, TWCOL402-10, EBCCH402-1, and HMCOC696-09). To enable bootstrap analysis, 4 data partitions were run separately: A) family Carabidae; B) family Dytiscidae; C) family Staphylinidae; and D) all other families together.

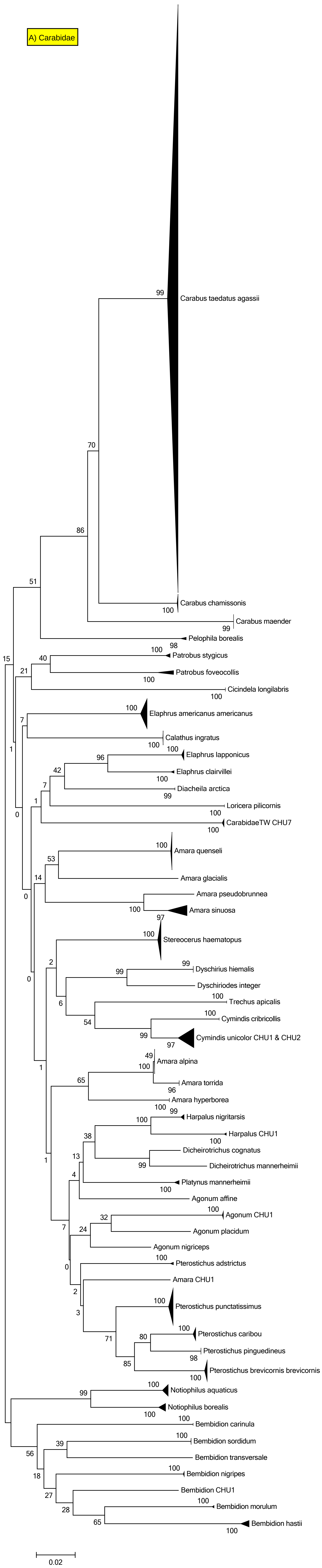
A) Carabidae
Carabus taedatus agassii
Carabus chamissonis
Carabus maender
Pelophila borealis
Patrobus stygicus
Patrobus foveocollis
Cicindela longilabris
Elaphrus americanus americanus
Calathus ingratus
Elaphrus lapponicus
Elaphrus clairvillei
Diacheila arctica
Loricera pilicornis
CarabidaeTW CHU7
Amara quenseli
Amara glacialis
Amara pseudobrunnea
Amara sinuosa
Stereocerus haematopus
Dyschirius hiemalis
Dyschiriodes integer
Trechus apicalis
Cymindis cribricollis
Cymindis unicolor CHU1 & CHU2
Amara alpina
Amara torrida
Amara hyperborea
Harpalus nigritarsis
Harpalus CHU1
Dicheirotrichus cognatus
Dicheirotrichus mannerheimii
Platynus mannerheimii
Agonum affine
Agonum CHU1
Agonum placidum
Agonum nigriceps
Pterostichus adstrictus
Amara CHU1
Pterostichus punctatissimus
Pterostichus caribou
Pterostichus pinguedineus
Pterostichus brevicornis brevicornis
Notiophilus aquaticus
Notiophilus borealis
Bembidion carinula
Bembidion sordidum
Bembidion transversale
Bembidion nigripes
Bembidion CHU1
Bembidion morulum
Bembidion hastii
0.02

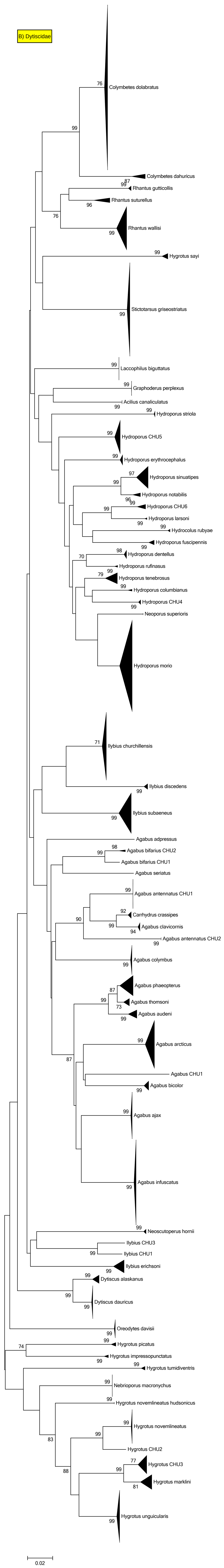

B) Dytiscidae
Colymbetes dolabratus
Colymbetes dahuricus
Rhantus gutticollis
Rhantus suturellus
Rhantus wallisi
Hygrotus sayi
Stictotarsus griseostriatus
Laccophilus biguttatus
Graphoderus perplexus
Acilius canaliculatus
Hydroporus striola
Hydroporus CHU5
Hydroporus erythrocephalus
Hydroporus sinuatipes
Hydroporus notabilis
Hydroporus CHU6
Hydroporus larsoni
Hydrocolus rubyae
Hydroporus fuscipennis
Hydroporus dentellus
Hydroporus rufinasus
Hydroporus tenebrosus
Hydroporus columbianus
Hydroporus CHU4
Neoporus superioris
Hydroporus morio
Ilybius churchillensis
Ilybius discedens
Ilybius subaeneus
Agabus adpressus
Agabus bifarius CHU2
Agabus bifarius CHU1
Agabus seriatus
Agabus antennatus CHU1
Carrhydrus crassipes
Agabus clavicornis
Agabus antennatus CHU2
Agabus colymbus
Agabus phaeopterus
Agabus thomsoni
Agabus audeni
Agabus arcticus
Agabus CHU1
Agabus bicolor
Agabus ajax
Agabus infuscatus
Neoscutoperus hornii
Ilybius CHU3
Ilybius CHU1
Ilybius erichsoni
Dytiscus alaskanus
Dytiscus dauricus
Oreodytes davisii
Hygrotus picatus
Hygrotus impressopunctatus
Hygrotus tumidiventris
Nebrioporus macronychus
Hygrotus novemlineatus hudsonicus
Hygrotus novemlineatus
Hygrotus CHU2
Hygrotus CHU3
Hygrotus marklini
Hygrotus unguicularis
0.02

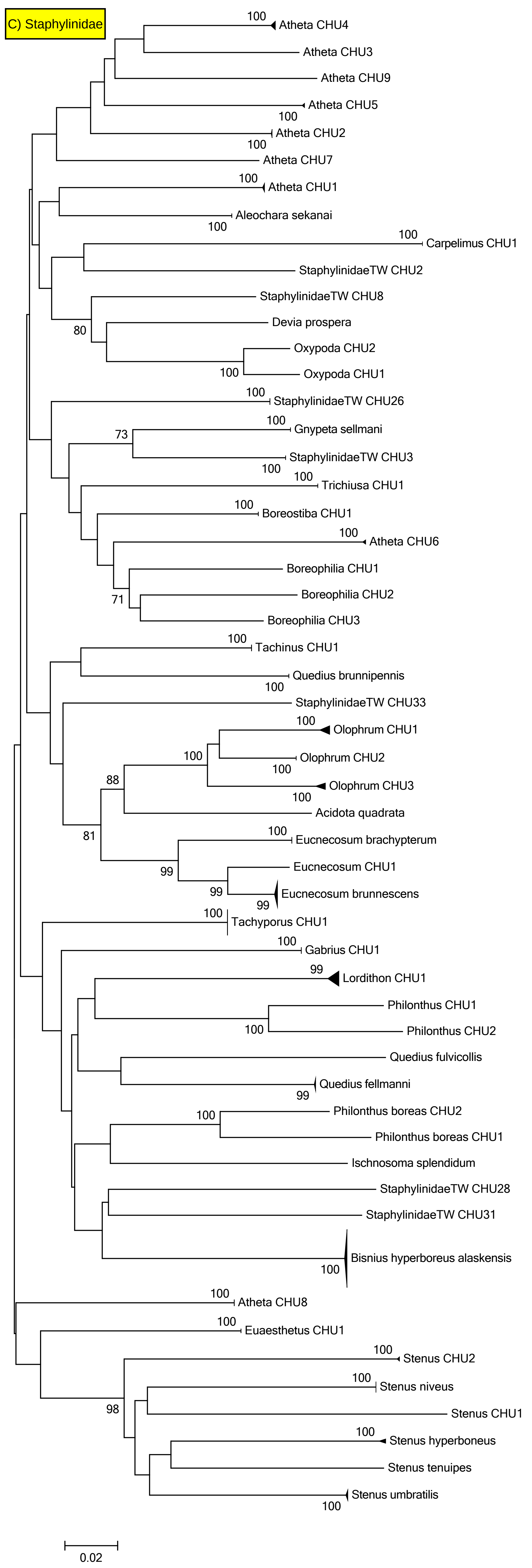
C) Staphylinidae
100
Atheta CHU4
Atheta CHU3
Atheta CHU9
Atheta CHU5
100
Atheta CHU2
100
Atheta CHU7
100
Atheta CHU1
Aleochara sekanai
100
100
Carpelimus CHU1
StaphylinidaeTW CHU2
StaphylinidaeTW CHU8
80
Devia prospera
Oxypoda CHU2
100
Oxypoda CHU1
100
StaphylinidaeTW CHU26
100
Gnypeta sellmani
73
StaphylinidaeTW CHU3
100
100
Trichiusa CHU1
100
Boreostiba CHU1
100
Atheta CHU6
Boreophilia CHU1
Boreophilia CHU2
71
Boreophilia CHU3
100
Tachinus CHU1
Quedius brunnipennis
100
StaphylinidaeTW CHU33
100
Olophrum CHU1
100
Olophrum CHU2
100
Olophrum CHU3
88
100
Acidota quadrata
81
100
Eucnecosum brachypterum
Eucnecosum CHU1
99
99
Eucnecosum brunnescens
99
100
Tachyporus CHU1
100
Gabrius CHU1
99
Lordithon CHU1
Philonthus CHU1
100
Philonthus CHU2
Quedius fulvicollis
Quedius fellmanni
99
100
Philonthus boreas CHU2
Philonthus boreas CHU1
Ischnosoma splendidum
StaphylinidaeTW CHU28
StaphylinidaeTW CHU31
Bisnius hyperboreus alaskensis
100
100
Atheta CHU8
100
Euaesthetus CHU1
100
Stenus CHU2
100
Stenus niveus
98
Stenus CHU1
100
Stenus hyperboneus
Stenus tenuipes
Stenus umbratilis
100
0.02

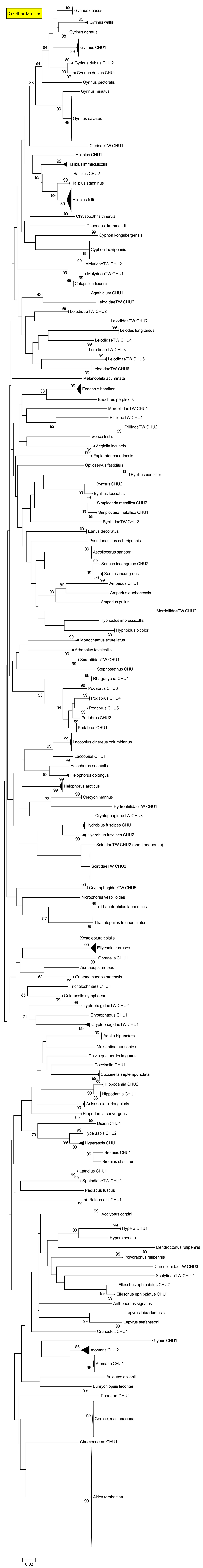

D) Other families
Gyrinus opacus
Gyrinus wallisi
Gyrinus aeratus
Gyrinus CHU1
Gyrinus dubius CHU2
Gyrinus dubius CHU1
Gyrinus pectoralis
Gyrinus minutus
Gyrinus cavatus
CleridaeTW CHU1
Haliplus CHU1
Haliplus immaculicollis
Haliplus CHU2
Haliplus stagninus
Haliplus falli
Chrysobothris trinervia
Phaenops drummondi
Cyphon kongsbergensis
Cyphon laevipennis
MelyridaeTW CHU2
MelyridaeTW CHU1
Catops luridipennis
Agathidium CHU1
LeiodidaeTW CHU2
LeiodidaeTW CHU8
LeiodidaeTW CHU7
Leiodes longitarsus
LeiodidaeTW CHU4
LeiodidaeTW CHU3
LeiodidaeTW CHU5
LeiodidaeTW CHU6
Melanophila acuminata
Enochrus hamiltoni
Enochrus perplexus
MordellidaeTW CHU1
PtiliidaeTW CHU1
PtiliidaeTW CHU2
Serica tristis
Aegialia lacustris
Explorator canadensis
Optioservus fastiditus
Byrrhus concolor
Byrrhus CHU2
Byrrhus fasciatus
Simplocaria metallica CHU2
Simplocaria metallica CHU1
ByrrhidaeTW CHU2
Eanus decoratus
Pseudanostirus ochreipennis
Ascoliocerus sanborni
Sericus incongruus CHU2
Sericus incongruus
Ampedus CHU1
Ampedus quebecensis
Ampedus pullus
MordellidaeTW CHU2
Hypnoidus impressicollis
Hypnoidus bicolor
Monochamus scutellatus
Arhopalus foveicollis
ScraptiidaeTW CHU1
Stephostethus CHU1
Rhagonycha CHU1
Podabrus CHU3
Podabrus CHU4
Podabrus CHU5
Podabrus CHU2
Podabrus CHU1
Laccobius cinereus columbianus
Laccobius CHU1
Helophorus orientalis
Helophorus oblongus
Helophorus arcticus
Cercyon marinus
HydrophilidaeTW CHU1
CryptophagidaeTW CHU3
Hydrobius fuscipes CHU1
Hydrobius fuscipes CHU2
ScirtidaeTW CHU2 (short sequence)
ScirtidaeTW CHU2
CryptophagidaeTW CHU5
Nicrophorus vespilloides
Thanatophilus lapponicus
Thanatophilus trituberculatus
Xestoleptura tibialis
Ellychnia corrusca
Ophraella CHU1
Acmaeops proteus
Gnathacmaeops pratensis
Tricholochmaea CHU1
Galerucella nymphaeae
CryptophagidaeTW CHU2
Cryptophagus CHU1
CryptophagidaeTW CHU1
Adalia bipunctata
Mulsantina hudsonica
Calvia quatuordecimguttata
Coccinella CHU1
Coccinella septempunctata
Hippodamia CHU2
Hippodamia CHU1
Anisosticta bitriangularis
Hippodamia convergens
Didion CHU1
Hyperaspis CHU2
Hyperaspis CHU1
Bromius CHU1
Bromius obscurus
Latridius CHU1
SphindidaeTW CHU1
Pediacus fuscus
Plateumaris CHU1
Acalyptus carpini
Hypera CHU1
Hypera seriata
Dendroctonus rufipennis
Polygraphus rufipennis
CurculionidaeTW CHU3
ScolytinaeTW CHU2
Ellescus ephippiatus CHU2
Ellescus ephippiatus CHU1
Anthonomus signatus
Lepyrus labradorensis
Lepyrus stefanssoni
Orchestes CHU1
Grypus CHU1
Atomaria CHU2
Atomaria CHU1
Auleutes epilobii
Euhrychiopsis lecontei
Phaedon CHU2
Gonioctena linnaeana
Chaetocnema CHU1
Altica tombacina
0.02
