# Supplementary material for: The diversity and biogeography of the Coleoptera of Churchill: insights from DNA barcoding
Source: BMC Ecol. 2013 Oct 29;13:40. doi: 10.1186/1472-6785-13-40 (PMC3819705; doi:10.1186/1472-6785-13-40)
Supplement: Additional file 4 — Genetic distances within and between 283 species or provisional species of Coleoptera of Churchill, based upon the 2972 specimens having barcode sequences of at least 500 bp. “N/A” for the maximum intraspecific distance indicates a sample size of just one specimen of sequence length of at least 500 bp for that species. Additional species or provisional species only represented by sequences of <500 bp occur in the list of specimens (Additional file 1). [file 1472-6785-13-40-S4.pdf]

Additional File 4. Genetic distances within and between 283 species or provisional species of Coleoptera of Churchill, based upon the 2972 specimens having barcode sequences of at least 500 bp. "N/A" for the maximum intraspecific distance indicates a sample size of just one specimen of sequence length of at least 500 bp for that species. Additional species or provisional species only represented by sequences of <500 bp occur in the list of specimens (Additional file 1).

Family	Species or Provisional Species	Maximum Intraspecific Distance	Nearest Neighbour Distance
Brachyceridae	*Grypus* CHU1	N/A	28.06
Buprestidae	*Chrysobothris trinervia*	1.4	12.9
Buprestidae	*Melanophila acuminata*	N/A	17.62
Buprestidae	*Phaenops drummondi*	N/A	15.38
Byrrhidae	ByrrhidaeTW CHU2	N/A	18.6
Byrrhidae	*Byrrhus* CHU2	N/A	2.8
Byrrhidae	*Byrrhus fasciatus*	0	2.8
Byrrhidae	*Simplocaria metallica* CHU1	0.61	2.49
Byrrhidae	*Simplocaria metallica* CHU2	N/A	2.49
Cantharidae	*Podabrus* CHU1	0.46	3.28
Cantharidae	*Podabrus* CHU2	0	3.13
Cantharidae	*Podabrus* CHU3	0	6.53
Cantharidae	*Podabrus* CHU4	0.16	4.23
Cantharidae	*Podabrus* CHU5	0.15	3.13
Cantharidae	*Rhagonycha* CHU1	0.15	12.75
Carabidae	*Agonum* CHU1	0.16	8.34
Carabidae	*Agonum affine*	N/A	9.85
Carabidae	*Agonum nigriceps*	N/A	8.76
Carabidae	*Agonum placidum*	N/A	8.76
Carabidae	*Amara* CHU1	N/A	9.04
Carabidae	*Amara alpina*	0.17	1.19
Carabidae	*Amara glacialis*	N/A	11.78
Carabidae	*Amara hyperborea*	0	8.01
Carabidae	*Amara pseudobrunnea*	N/A	4.4
Carabidae	*Amara quenseli*	0.49	10.33
Carabidae	*Amara sinuosa*	2.13	4.4
Carabidae	*Amara torrida*	0	1.19
Carabidae	*Bembidion* CHU1	N/A	12.72
Carabidae	*Bembidion carinula*	N/A	13.67
Carabidae	*Bembidion hastii*	1.02	12.4
Carabidae	*Bembidion morulum*	0.18	12.4
Carabidae	*Bembidion nigripes*	0.32	12.58

Carabidae	*Bembidion sordidum*	0.16	12.51
Carabidae	*Bembidion transversale*	N/A	12.83
Carabidae	*Calathus ingratus*	0.34	12.31
Carabidae	CarabidaeTW CHU7	0.32	12.07
Carabidae	*Carabus chamissonis*	0.51	7.33
Carabidae	*Carabus maender*	0.16	12.69
Carabidae	*Carabus taedatus agassii*	1.56	7.33
Carabidae	*Cicindela longilabris*	0	15.68
Carabidae	*Cymindis cribricollis*	0	5.04
Carabidae	*Cymindis unicolor* CHU1	1.62	4.62
Carabidae	*Cymindis unicolor* CHU2	N/A	4.62
Carabidae	*Diacheila arctica*	N/A	10.96
Carabidae	*Dicheirotrichus cognatus*	N/A	4.57
Carabidae	*Dicheirotrichus mannerheimii*	N/A	4.57
Carabidae	*Dyschiriodes integer*	N/A	7.95
Carabidae	*Dyschirius hiemalis*	0.32	7.95
Carabidae	*Elaphrus americanus americanus*	1.39	12.01
Carabidae	*Elaphrus clairvillei*	0.3	7.01
Carabidae	*Elaphrus lapponicus*	0.35	7.01
Carabidae	*Harpalus* CHU1	0.2	5.57
Carabidae	*Harpalus nigritarsis*	0.46	5.57
Carabidae	*Loricera pilicornis*	0	14.2
Carabidae	*Notiophilus aquaticus*	0.78	7.67
Carabidae	*Notiophilus borealis*	0.77	7.67
Carabidae	*Patrobus foveocollis*	1.7	12.39
Carabidae	*Patrobus stygicus*	0.46	12.39
Carabidae	*Pelophila borealis*	N/A	12.88
Carabidae	*Platynus mannerheimii*	0.49	7.91
Carabidae	*Pterostichus adstrictus*	0.31	8.59
Carabidae	*Pterostichus brevicornis brevicornis*	0.32	5.97
Carabidae	*Pterostichus caribou*	2.36	3.86
Carabidae	*Pterostichus pinguedineus*	0	3.86
Carabidae	*Pterostichus punctatissimus*	1.15	6.77
Carabidae	*Stereocerus haematopus*	0.66	9.62
Carabidae	*Trechus apicalis*	0	11.62
Cerambycidae	*Acmaeops proteus*	N/A	9.63
Cerambycidae	*Arhopalus foveicollis*	0.92	14.19
Cerambycidae	*Gnathacmaeops pratensis*	0.31	9.63
Cerambycidae	*Monochamus scutellatus*	1.08	14.19
Cerambycidae	*Xestoleptura tibialis*	N/A	16.91
Chrysomelidae	*Altica tombacina*	2.4	17.22
Chrysomelidae	*Bromius* CHU1	N/A	2.82

Chrysomelidae	*Bromius obscurus*	N/A	2.82
Chrysomelidae	*Chaetocnema* CHU1	N/A	17.01
Chrysomelidae	*Galerucella nymphaeae*	0.16	11.64
Chrysomelidae	*Gonioctena linnaeana*	0.61	18.34
Chrysomelidae	*Phaedon* CHU2	N/A	18.53
Chrysomelidae	*Plateumaris* CHU1	1.57	16.88
Chrysomelidae	*Tricholochmaea* CHU1	N/A	11.64
Cleridae	CleridaeTW CHU1	N/A	14.49
Coccinellidae	*Adalia bipunctata*	2.27	15.18
Coccinellidae	*Anisosticta bitriangularis*	0.78	15.3
Coccinellidae	*Calvia quatuordecimguttata*	N/A	12.6
Coccinellidae	*Coccinella* CHU1	N/A	12.6
Coccinellidae	*Coccinella septempunctata*	0.62	13.69
Coccinellidae	*Didion* CHU1	0.15	15.11
Coccinellidae	*Hippodamia* CHU1	0.31	2.17
Coccinellidae	*Hippodamia* CHU2	0.62	2.17
Coccinellidae	*Hippodamia convergens*	0	15.54
Coccinellidae	*Hyperaspis* CHU1	1.7	3.76
Coccinellidae	*Hyperaspis* CHU2	0	3.76
Coccinellidae	*Mulsantina hudsonica*	N/A	14.06
Cryptophagidae	*Atomaria* CHU1	0.77	8.48
Cryptophagidae	*Atomaria* CHU2	2.01	8.48
Cryptophagidae	CryptophagidaeTW CHU1	N/A	15.53
Cryptophagidae	CryptophagidaeTW CHU2	0.16	15.53
Cryptophagidae	CryptophagidaeTW CHU5	N/A	17.83
Cryptophagidae	*Cryptophagus* CHU1	N/A	15.79
Cucujidae	*Pediacus fuscus*	N/A	16.61
Curculionidae	*Acalyptus carpini*	0.46	18.51
Curculionidae	*Anthonomus signatus*	N/A	20.09
Curculionidae	*Auleutes epilobii*	N/A	17.6
Curculionidae	*Elleschus ephippiatus* CHU1	0.47	2.43
Curculionidae	*Elleschus ephippiatus* CHU2	N/A	2.43
Curculionidae	*Euhrychiopsis lecontei*	0.6	17.6
Curculionidae	*Hypera* CHU1	0.46	10.99
Curculionidae	*Hypera seriata*	N/A	10.99
Curculionidae	*Lepyrus labradorensis*	N/A	10.25
Curculionidae	*Lepyrus stefanssoni*	0.16	10.25
Curculionidae	*Orchestes* CHU1	N/A	17.82
Curculionidae	*Polygraphus rufipennis*	0.31	20.57
Dytiscidae	*Acilius canaliculatus*	0	11.67
Dytiscidae	*Agabus* CHU1	N/A	10.75
Dytiscidae	*Agabus adpressus*	N/A	11.56
Dytiscidae	*Agabus ajax*	0.46	7.8

Dytiscidae	*Agabus antennatus* CHU1	0.33	5.96
Dytiscidae	*Agabus antennatus* CHU2	0	8.92
Dytiscidae	*Agabus arcticus*	1.57	9.63
Dytiscidae	*Agabus audeni*	1.71	3.12
Dytiscidae	*Agabus bicolor*	0.92	8.63
Dytiscidae	*Agabus bifarius* CHU1	N/A	2.39
Dytiscidae	*Agabus bifarius* CHU2	N/A	2.39
Dytiscidae	*Agabus clavicornis*	0.3	2.71
Dytiscidae	*Agabus colymbus*	0.81	10.6
Dytiscidae	*Agabus infuscatus*	1.44	7.8
Dytiscidae	*Agabus phaeopterus*	2.83	1.15
Dytiscidae	*Agabus seriatus*	N/A	9.86
Dytiscidae	*Agabus thomsoni*	1.08	1.15
Dytiscidae	*Carrhydrus crassipes*	0.98	2.71
Dytiscidae	*Colymbetes dahuricus*	1.08	7.56
Dytiscidae	*Colymbetes dolabratus*	3.76	7.56
Dytiscidae	*Dytiscus alaskanus*	1.14	3.16
Dytiscidae	*Dytiscus dauricus*	0.62	3.16
Dytiscidae	*Graphoderus perplexus*	0.72	11.22
Dytiscidae	*Hydrocolus rubyae*	N/A	11.77
Dytiscidae	*Hydroporus* CHU4	0.46	5.38
Dytiscidae	*Hydroporus* CHU5	1.86	8.42
Dytiscidae	*Hydroporus* CHU6	1.39	5.21
Dytiscidae	*Hydroporus columbianus*	0.62	6.79
Dytiscidae	*Hydroporus dentellus*	1.08	5.13
Dytiscidae	*Hydroporus erythrocephalus*	0.66	8.59
Dytiscidae	*Hydroporus fuscipennis*	1.09	10.56
Dytiscidae	*Hydroporus larsoni*	0.3	5.21
Dytiscidae	*Hydroporus morio*	3.46	6.37
Dytiscidae	*Hydroporus sinuatipes*	2.81	2.93
Dytiscidae	*Hydroporus notabilis*	1.25	2.93
Dytiscidae	*Hydroporus rufinasus*	0.46	5.13
Dytiscidae	*Hydroporus striola*	0.31	11.59
Dytiscidae	*Hydroporus tenebrosus*	1.54	5.38
Dytiscidae	*Hygrotus* CHU2	N/A	3.6
Dytiscidae	*Hygrotus* CHU3	2.49	2.2
Dytiscidae	*Hygrotus impressopunctatus*	0.66	13.01
Dytiscidae	*Hygrotus marklini*	1.55	2.2
Dytiscidae	*Hygrotus novemlineatus hudsonicus*	N/A	11.08
Dytiscidae	*Hygrotus novemlineatus*	0.81	3.6
Dytiscidae	*Hygrotus picatus*	0.61	13.29
Dytiscidae	*Hygrotus sayi*	0.8	15.45

Dytiscidae	*Hygrotus unguicularis*	1.11	7.66
Dytiscidae	*Ilybius* CHU1	N/A	4.15
Dytiscidae	*Ilybius churchillensis*	1.29	9.03
Dytiscidae	*Ilybius* CHU3	N/A	4.15
Dytiscidae	*Ilybius discedens*	0.76	9.03
Dytiscidae	*Ilybius erichsoni*	2.02	12.01
Dytiscidae	*Ilybius subaeneus*	4.08	11.4
Dytiscidae	*Laccophilus biguttatus*	0.17	11.1
Dytiscidae	*Nebrioporus macronychus*	0.37	11.79
Dytiscidae	*Neoscutoperus hornii*	0.3	14.3
Dytiscidae	*Oreodytes davisii*	0.31	13.55
Dytiscidae	*Rhantus gutticollis*	0.62	8.23
Dytiscidae	*Rhantus suturellus*	2.66	6.71
Dytiscidae	*Rhantus wallisi*	2.42	6.71
Dytiscidae	*Stictotarsus griseostriatus*	1.11	12.52
Elateridae	*Ampedus* CHU1	0.48	12.84
Elateridae	*Ampedus pullus*	N/A	13.86
Elateridae	*Ampedus quebecensis*	N/A	12.84
Elateridae	*Ascoliocerus sanborni*	0.49	15.25
Elateridae	*Eanus decoratus*	0.35	17.59
Elateridae	*Hypnoidus bicolor*	0.34	12.83
Elateridae	*Hypnoidus impressicollis*	0	12.83
Elateridae	*Pseudanostirus ochreipennis*	N/A	16.49
Elateridae	*Sericus incongruus*	0.92	2.32
Elateridae	*Sericus incongruus* CHU2	0.15	2.32
Elmidae	*Optioservus fastiditus*	N/A	17.99
Gyrinidae	*Gyrinus* CHU1	1.38	6.2
Gyrinidae	*Gyrinus aeratus*	0.17	3.31
Gyrinidae	*Gyrinus cavatus*	1.15	3.44
Gyrinidae	*Gyrinus dubius* CHU1	0.92	1.7
Gyrinidae	*Gyrinus dubius* CHU2	0.69	1.7
Gyrinidae	*Gyrinus minutus*	N/A	3.44
Gyrinidae	*Gyrinus opacus*	0.81	3.31
Gyrinidae	*Gyrinus pectoralis*	N/A	8.45
Gyrinidae	*Gyrinus wallisi*	0.49	4.77
Haliplidae	*Haliplus* CHU1	N/A	10
Haliplidae	*Haliplus* CHU2	N/A	7.95
Haliplidae	*Haliplus falli*	1.55	3.44
Haliplidae	*Haliplus immaculicollis*	1.55	7.92
Haliplidae	*Haliplus stagninus*	0.16	3.44
Heteroceridae	*Explorator canadensis*	0.32	19.9
Hydrophilidae	*Cercyon marinus*	0	15.06
Hydrophilidae	*Enochrus hamiltoni*	1.86	15.5

Hydrophilidae	*Helophorus arcticus*	1.08	11.75
Hydrophilidae	*Helophorus oblongus*	1.23	6.06
Hydrophilidae	*Helophorus orientalis*	N/A	6.06
Hydrophilidae	*Hydrobius fuscipes* CHU1	1.08	5.88
Hydrophilidae	*Hydrobius fuscipes* CHU2	1.47	5.88
Hydrophilidae	*Laccobius* CHU1	0.49	5.74
Hydrophilidae	*Laccobius cinereus columbianus*	0.62	5.74
Lampyridae	*Ellychnia corrusca*	2.65	18.38
Latridiidae	*Latridius* CHU1	0.79	16.01
Leiodidae	*Agathidium* CHU1	N/A	15.35
Leiodidae	*Catops luridipennis*	0.16	16.49
Leiodidae	*Leiodes longitarsus*	0.16	18.2
Leiodidae	LeiodidaeTW CHU2	N/A	15.35
Leiodidae	LeiodidaeTW CHU3	N/A	17.76
Leiodidae	LeiodidaeTW CHU5	0.51	16.68
Leiodidae	LeiodidaeTW CHU6	N/A	16.68
Leiodidae	LeiodidaeTW CHU7	N/A	16.17
Leiodidae	LeiodidaeTW CHU8	0.77	15.05
Melyridae	MelyridaeTW CHU1	0.3	4.93
Melyridae	MelyridaeTW CHU2	0.15	4.93
Ptiliidae	PtiliidaeTW CHU1	N/A	22.27
Scarabaeidae	*Aegialia lacustris*	0.46	16.73
Scirtidae	*Cyphon kongsbergensis*	0.17	16.82
Scirtidae	*Cyphon laevipennis*	0.77	17.55
Scirtidae	ScirtidaeTW CHU2	1.26	17.98
Scraptiidae	ScraptiidaeTW CHU1	0.32	16.84
Silphidae	*Nicrophorus vespilloides*	N/A	17.57
Silphidae	*Thanatophilus lapponicus*	1.08	14.32
Silphidae	*Thanatophilus trituberculatus*	0.64	14.32
Sphindidae	SphindidaeTW CHU1	0.35	15.74
Staphylinidae	*Acidota quadrata*	N/A	12.93
Staphylinidae	*Aleochara sekanai*	0	12.42
Staphylinidae	*Atheta* CHU1	0.15	13.89
Staphylinidae	*Atheta* CHU2	0.16	12.07
Staphylinidae	*Atheta* CHU3	N/A	12.07
Staphylinidae	*Atheta* CHU4	0	12.22
Staphylinidae	*Atheta* CHU5	0.16	14.03
Staphylinidae	*Atheta* CHU6	N/A	14.41
Staphylinidae	*Atheta* CHU7	N/A	15.67
Staphylinidae	*Atheta* CHU8	0	13.33
Staphylinidae	*Atheta* CHU9	N/A	14.92
Staphylinidae	*Bisnius hyperboreus alaskensis*	0.73	17.8
Staphylinidae	*Boreophilia* CHU1	N/A	10.2

Staphylinidae	*Boreophilia* CHU2	N/A	10.63
Staphylinidae	*Boreophilia* CHU3	N/A	10.2
Staphylinidae	*Boreostiba* CHU1	0	12.74
Staphylinidae	*Carpelimus* CHU1	N/A	17.84
Staphylinidae	*Devia prospera*	N/A	12.91
Staphylinidae	*Euaesthetus* CHU1	0	12.79
Staphylinidae	*Eucnecosum* CHU1	N/A	4.36
Staphylinidae	*Eucnecosum brachypterum*	0	7.16
Staphylinidae	*Eucnecosum brunnescens*	1.46	4.36
Staphylinidae	*Gabrius* CHU1	0	16.77
Staphylinidae	*Ischnosoma splendidum*	N/A	16.91
Staphylinidae	*Lordithon* CHU1	1.46	17.29
Staphylinidae	*Olophrum* CHU1	0.77	6.27
Staphylinidae	*Olophrum* CHU2	0	6.27
Staphylinidae	*Olophrum* CHU3	0.77	7.76
Staphylinidae	*Oxypoda* CHU1	N/A	3.89
Staphylinidae	*Oxypoda* CHU2	N/A	3.89
Staphylinidae	*Philonthus* CHU1	N/A	9.45
Staphylinidae	*Philonthus* CHU2	N/A	9.45
Staphylinidae	*Philonthus boreas* CHU1	N/A	9.88
Staphylinidae	*Philonthus boreas* CHU2	N/A	9.88
Staphylinidae	*Quedius brunnipennis*	0	14.18
Staphylinidae	*Quedius fellmanni*	0.15	15
Staphylinidae	*Quedius fulvicollis*	N/A	17.32
Staphylinidae	StaphylinidaeTW CHU2	N/A	13.02
Staphylinidae	StaphylinidaeTW CHU28	N/A	16.81
Staphylinidae	StaphylinidaeTW CHU31	N/A	17.17
Staphylinidae	StaphylinidaeTW CHU3	0	14.37
Staphylinidae	StaphylinidaeTW CHU33	N/A	15.86
Staphylinidae	StaphylinidaeTW CHU8	N/A	12.91
Staphylinidae	*Stenus* CHU1	N/A	19.83
Staphylinidae	*Stenus* CHU2	0.15	18.8
Staphylinidae	*Stenus hyperboneus*	0.46	15.36
Staphylinidae	*Stenus niveus*	0	16.63
Staphylinidae	*Stenus tenuipes*	N/A	16.12
Staphylinidae	*Stenus umbratilis*	0.15	15.36
Staphylinidae	*Tachinus* CHU1	0	13.36
Staphylinidae	*Tachyporus* CHU1	0.16	13.02
**AVERAGE**		**0.75**	**10.4**
