# Supplementary material for: The diversity and biogeography of the Coleoptera of Churchill: insights from DNA barcoding
Source: BMC Ecol. 2013 Oct 29;13:40. doi: 10.1186/1472-6785-13-40 (PMC3819705; doi:10.1186/1472-6785-13-40)
Supplement: Additional file 5 — Summary of collection habitats for specimens with species determinations in the Churchill Coleoptera barcode library. [file 1472-6785-13-40-S5.pdf]

Additional File 5. Summary of collection habitats for specimens with species determinations in the Churchill Coleoptera barcode library.

Species	Collected Habitats in Churchill Region*
**Buprestidae**	
Chrysobothris trinervia	Rocky seashore (1), spruce burn (1)
Melanophila fulvoguttata	Town (1)
Melanophila acuminata	Spruce forest (1)
**Byrrhidae**	
Byrrhus concolor	Rocky seashore (2), tundra (2)
Byrrhus fasciatus	Tundra lakeshore (1), town (1)
Simplocaria metallica	Sandy seashore (2), forested lakeshore (2)
**Carabidae**	
Agonum affine	Tundra lakeshore (1)
Agonum nigriceps	Spruce burn pond (1)
Agonum placidum	Tundra lakeshore (1)
Amara alpina	Tundra (7), riparian (4), rocky seashore (3), spruce burn (2), other (3)
Amara glacialis	Sandy seashore (1)
Amara hyperborea	Sandy seashore (2), tundra lakeshore (1)
Amara pseudobrunnea	Tundra (1)
Amara quenseli	Sandy seashore (11), tundra (7), town (4), riparian (3), forested lakeshore (3), rocky seashore (1)
Amara sinuosa	Tundra (5), forested lakeshore (3)
Amara torrida	Town (2), sandy seashore (1), town (1)
Bembidion carinula	Forested lakeshore (1), Churchill R. riparian (1)
Bembidion hastii	Churchill R. riparian (2), tundra (2), sandy seashore (1)
Bembidion morulum	Riparian (1), rocky seashore (1)
Bembidion nigripes	Churchill R. riparian (3), town (1)
Bembidion sordidum	Churchill R. riparian (4), tundra (1)
Bembidion transversale	Forested lakeshore (1)
Calathus ingratus	Churchill R. riparian (4), tundra (4), forested lakeshore (2), open wetland (1)

Carabus chamissonis	Tundra (6), krummholz (3), ditch (3), sandy seashore (1), open wetland (1)
Carabus maender	Churchill R. riparian (5), rocky seashore (3), ditch (2), open wetland (1)
Carabus taedatus agassii	Sandy seashore (233), forested lakeshore (61), krummholz (61), tundra (48), other (47)
Cicindela longilabris	Open sand (3)
Cymindis cribricollis	Forested lakeshore (2)
Cymindis unicolor	Riparian (6), krummholz (5), tundra (3), bluff pond (1)
Diacheila arctica	Riparian (2)
Dicheirotrichus cognatus	Spruce forest (1)
Dicheirotrichus mannerheimii	Town (1)
Dyschiriodes integer	Tundra (1)
Dyschirius hiemalis	Rocky seashore (3), krummholz (2)
Elaphrus americanus americanus	Churchill R. riparian (21), open wetland (2)
Elaphrus clairvillei	Churchill R. riparian (1), tundra (1)
Elaphrus lapponicus	Open wetland (3), rocky seashore (2), riparian (1), tundra (1), open wetland (1)
Harpalus nigritarsis	Tundra (3), tundra lakeshore (1)
Loricera pilicornis	Churchill R. riparian (1), tundra (1)
Notiophilus aquaticus	Forested lakeshore (7), riparian (1), tundra (1)
Notiophilus borealis	Tundra (4), sandy seashore (1), krummholz (1)
Patrobus foveocollis	Churchill R. riparian (3)
Patrobus stygicus	Churchill R. riparian (1), rocky seashore (1), tundra (1)
Pelophila borealis	Churchill R. riparian (2)
Platynus mannerheimii	Open wetland (2), tundra (1)
Pterostichus adstrictus	Tundra (2)
Pterostichus brevicornis brevicornis	Tundra (6), town (4), forested lakeshore (2), tundra lakeshore (2), other (2)
Pterostichus caribou	Tundra (4), riparian (3), sandy seashore (3)
Pterostichus pinguedineus	Rocky seashore (3), sandy seashore (1), tundra (1)
Pterostichus punctatissimus	Tundra (10), open wetland (5), rocky seashore (3), tundra lakeshore (2), other (8)
Stereocerus haematopus	Krummholz (16), riparian (6), rocky seashore (3), forested lakeshore (3), other (3)
Trechus apicalis	Churchill R. riparian (2)

**Cerambycidae**	
Acmaeops proteus	Spruce forest (1)
Arhopalus foveicollis	Forested lakeshore (2), Churchill R. riparian (1)
Gnathacmaeops pratensis	Open wetland (2), krummholz (1), spruce burn (1)
Monochamus scutellatus	Open wetland (1), riparian (1), tundra (1)
Xestoleptura tibialis	Spruce forest (1)
**Chrysomelidae**	
Altica tombacina	Rocky seashore (48), spruce burn (46), tundra lakeshore (21), forested lakeshore (17), other (2)
Bromius obscurus	Spruce burn (1)
Galerucella nymphaeae	Tundra (2)
Gonioctena linnaeana	Willow thicket (17), bare ground (11), spruce burn (7), riparian (6), other (7)
**Coccinellidae**	
Adalia bipunctata	Spruce burn (11), rocky seashore (2), tundra (2), willow thicket (1)
Anisosticta bitriangularis	Rocky seashore (3), spruce burn (2), other (4)
Calvia quatuordecimguttata	Churchill R. riparian (1)
Coccinella septempunctata	Tundra (6), other (4)
Hippodamia convergens	Tundra (2), spruce burn (1)
Mulsantina hudsonica	Willow thicket (1)
**Cucujidae**	
Pediacus fuscus	Spruce burn (1)
**Curculionidae**	
Acalyptus carpini	Spruce burn (5), open wetland (4), tundra lakeshore (4), tundra (3), other (8)
Anthonomus signatus	Forested lakeshore (1)
Auleutes epilobii	Spruce burn (1)
Dendroctonus rufipennis	Spruce burn (2)
Elleschus ephippiatus	Tundra (4)
Euhrychiopsis lecontei	Churchill R. riparian (1), forested lakeshore (1)

Hypera seriata	Rocky seashore (1)
Lepyrus labradorensis	Town (1)
Lepyrus stefanssoni	Town (2)
Polygraphus rufipennis	Spruce forest (2)
**Dytiscidae**	
Acilius canaliculatus	Tundra pond (3), bluff pond (1)
Agabus adpressus	Spruce burn pond (1)
Agabus ajax	Tundra pond (24), bluff pond (10), Churchill R. (6), river/stream (6), other (5)
Agabus antennatus	Tundra pond (15), Churchill R. (7), spruce burn pond (6), bluff pond (4), other (3)
Agabus arcticus	Churchill R. (21), tundra pond (11), river/stream (7), other (13)
Agabus audeni	Tundra pond (5), Churchill R. (2), other (2)
Agabus bicolor	Bluff pond (8), other (2)
Agabus bifarius	Bluff pond (1), spruce burn pond (1), tundra pond (1)
Agabus clavicornis	Bluff pond (5), Churchill R. (2), spruce burn pond (2)
Agabus colymbus	Bluff pond (23), tundra pond (5), Churchill R. (4)
Agabus infuscatus	Bluff pond (83), Churchill R. (4), tundra pond (3), other (3)
Agabus seriatus	Spruce burn pond (1)
Agabus phaeopterus	Churchill R. (9), river/stream (5), tundra pond (3), other (5)
Agabus thomsoni	River/stream (6), ditch (1), tundra pond (1)
Carrhydrus crassipes	Tundra pond (3), bluff pond (2), other (2)
Colymbetes dahuricus	Ditch (3), tundra pond (2)
Colymbetes dolabratus	Bluff pond (82), tundra pond (45), river/stream (14), lake (8), spruce forest pond (8), spruce burn pond (7), other (16)
Dytiscus alaskanus	Tundra pond (6), river/stream (2), bluff pond (1)
Dytiscus dauricus	Bluff pond (19), tundra pond (11), spruce burn pond (2), river/stream (2), other (2)
Graphoderus perplexus	Tundra pond (9), bluff pond (3), spruce forest pond (2), other (2)
Hydrocolus rubyae	Tundra pond (3)
Hydroporus columbianus	Spruce forest pond (1), tundra pond (1)
Hydroporus dentellus	Tundra pond (4), bluff pond (2), ditch (2), spruce burn pond (1)
Hydroporus erythrocephalus	Tundra pond (5), river/stream (3), Churchill R. (2), spruce burn pond (1)
Hydroporus fuscipennis	Tundra pond (3), bluff pond (1), town (1)

Hydroporus larsoni	Tundra pond (2)
Hydroporus morio	Bluff pond (97), tundra pond (4)
Hydroporus notabilis	Bluff pond (3), tundra pond (1)
Hydroporus rufinasus	Tundra pond (2), bluff pond (1)
Hydroporus sinuatipes	Bluff pond (15), tundra pond (7), river/stream (2)
Hydroporus striola	Tundra pond (3), bluff pond (2), Churchill R. (1)
Hydroporus tenebrosus	Bluff pond (6), tundra lakeshore (2), river/stream (2), other (2)
Hygrotus impressopunctatus	Bluff pond (2), tundra pond (1)
Hygrotus marklini	Bluff pond (7), tundra pond (5), spruce forest pond (3), river/stream (1)
Hygrotus novemlineatus	Bluff pond (35), tundra pond (2)
Hygrotus picatus	Tundra pond (2), spruce forest pond (1), tundra pond (1), open wetland (1)
Hygrotus sayi	Spruce burn pond (3), tundra pond (2), Churchill R. (1)
Hygrotus tumidiventris	Tundra pond (4)
Hygrotus unguicularis	Tundra pond (27), Churchill R. (10), Churchill R. riparian (9), bluff pond (7), other (4)
Ilybius discedens	Tundra pond (3), ditch (2), bluff pond (1)
Ilybius erichsoni	Spruce forest pond (4), Churchill R. (4), bluff pond (3), tundra pond (2), ditch (1)
Ilybius subaeneus	Tundra pond (18), spruce forest pond (6), river/stream (6), Churchill R. riparian (5), other (9)
Laccophilus biguttatus	Tundra pond (17), spruce forest pond (4), bluff pond (4)
Nebrioporus macronychus	Churchill R. (15), river/stream (5), Churchill R. riparian (2), tundra pond (2)
Neoporus superioris	Tundra pond (1)
Neoscutoperus hornii	Ditch (3), spruce burn (2)
Oreodytes davisii	Churchill R. (13), lake (4), river/stream (2), tundra pond (1)
Rhantus gutticollis	Bluff pond (4), krummholz (1)
Rhantus suturellus	Bluff pond (3), open wetland (1), spruce burn pond (1)
Rhantus wallisi	Tundra pond (19), bluff pond (16), spruce forest pond (4), spruce burn pond (3), other (5)
Stictotarsus griseostriatus	Bluff pond (41), tundra pond (30), rocky seashore (8), tundra lakeshore (7), other (16)
**Elateridae**	
Ampedus pullus	Spruce forest (1)
Ampedus quebecensis	Tundra (1)
Ascoliocerus sanborni	Town (7), rocky seashore (5), tundra (2), riparian (2), sandy seashore (1)
Eanus decoratus	Spruce burn (3), forested lakeshore (2), riparian (2), spruce forest (1)

Hypnoidus bicolor	Forested lakeshore (4), tundra (2), other (4)
Hypnoidus impressicollis	Rocky seashore (7), tundra (2), Churchill R. riparian (1)
Pseudanostirus ochreipennis	Spruce burn (1)
Sericus incongruus	Riparian (2), spruce burn (2), other (4)
**Elmidae**	
Optioservus fastiditus	Churchill R. (1)
**Gyrinidae**	
Gyrinus aeratus	Spruce forest pond (4), river/stream (2), spruce burn pond (1), lake (1)
Gyrinus cavatus	Tundra pond (38), bluff pond (7), open wetland (5), lake (4), other (6)
Gyrinus dubius	Churchill R. (2), spruce burn pond (2), bluff pond (1), tundra pond (1)
Gyrinus minutus	Tundra pond (1)
Gyrinus opacus	Bluff pond (6), tundra pond (5), lake (3), other (3)
Gyrinus pectoralis	Open wetland (1)
Gyrinus wallisi	Churchill R. (2), lake (1), tundra pond (1)
**Haliplidae**	
Haliplus falli	Bluff pond (17), Churchill R. (6), ditch (4), river/stream (2), tundra pond (1)
Haliplus immaculicollis	Churchill R. (2), other (4)
Haliplus stagninus	Bluff pond (5), tundra pond (1)
**Heteroceridae**	
Explorator canadensis	Town (3), Churchill R. riparian (2)
**Hydrophilidae**	
Cercyon marinus	Churchill R. riparian (3), tundra (1), town (1)
Enochrus hamiltoni	Spruce burn pond (3), tundra pond (3), other (9)
Enochrus perplexus	Rocky seashore (1)
Helophorus arcticus	Sandy seashore (6), lake (6), bluff pond (2), Churchill R. riparian (1)
Helophorus oblongus	Tundra pond (4)
Helophorus orientalis	Bluff pond (1)

Hydrobius fuscipes	Tundra pond (6), bluff pond (4), Churchill R. (2), other (4)
Laccobius cinereus columbianus	Tundra pond (15), bluff pond (5), spruce burn pond (2), river/stream (2)
**Lampyridae**	
Ellychnia corrusca	Open wetland (4), spruce burn (3), other (6)
**Leiodidae**	
Catops luridipennis	Spruce burn (2), tundra (2), other (2)
Leiodes longitarsus	Forested lakeshore (8)
**Scarabaeidae**	
Aegialia lacustris	Churchill R. riparian (2), tundra (1)
Serica tristis	Forested lakeshore (1)
**Scirtidae**	
Cyphon kongsbergensis	Tundra (3), open sand (1)
Cyphon laevipennis	Churchill R. riparian (10), tundra pond (5), other (9)
**Silphidae**	
Nicrophorus vespilloides	Churchill R. riparian (1)
Thanatophilus lapponicus	Tundra (4), krummholz (1)
Thanatophilus trituberculatus	Churchill R. riparian (17), sandy seashore (6), rocky seashore (2), open sand (2), other (2)
**Staphylinidae**	
Acidota quadrata	Tundra (1)
Aleochara sekanai	Churchill R. riparian (1), tundra (1)
Bisnius hyperboreus alaskensis	Sandy seashore (16), rocky seashore (8), open sand (3)
Devia prospera	Forested lakeshore (1)
Eucnecosum brachypterum	Rocky seashore (2), sandy seashore (1)
Eucnecosum brunnescens	Forested lakeshore (7), town (3), other (3)

Gnypeta sellmani	Tundra (2)
Ischnosoma splendidum	Forested lakeshore (1)
Philonthus boreas	Churchill R. riparian (1), town (1)
Quedius brunnipennis	Spruce forest (1), spruce burn (1)
Quedius fellmanni	Town (3), Churchill R. riparian (1), riparian (1), open wetland (1),
Quedius fulvicollis	Spruce forest (1)
Stenus hyperboneus	Spruce forest (2)
Stenus niveus	Tundra lakeshore (2), forested lakeshore (1), sandy lakeshore (1), town (1)
Stenus tenuipes	Riparian (1)
Stenus umbratilus	Open wetland (3), tundra lakeshore (2)

* this study only; number of barcoded specimens in parentheses. "Town" refers to the Town of Churchill. For those with multiple collected habitats, habitats of single specimens indicated as "other"
